# Multipopulation mortality modelling and forecasting: the weighted multivariate functional principal component approaches

**DOI:** 10.1080/02664763.2022.2104228

**Published:** 2022-08-03

**Authors:** Ka Kin Lam, Bo Wang

**Affiliations:** School of Mathematics and Actuarial Science, University of Leicester, Leicester, UK

**Keywords:** Mortality modelling, coherent forecasts, functional principal component analysis, multivariate functional data analysis, Lee–Carter model, product-ratio model

## Abstract

Human mortality patterns and trajectories in closely related populations are likely linked together and share similarities. It is always desirable to model them simultaneously while taking their heterogeneity into account. This article introduces two new models for joint mortality modelling and forecasting multiple subpopulations using the multivariate functional principal component analysis techniques. The first model extends the independent functional data model to a multipopulation modelling setting. In the second one, we propose a novel multivariate functional principal component method for coherent modelling. Its design primarily fulfils the idea that when several subpopulation groups have similar socio-economic conditions or common biological characteristics such close connections are expected to evolve in a non-diverging fashion. We demonstrate the proposed methods by using sex-specific mortality data. Their forecast performances are further compared with several existing models, including the independent functional data model and the Product-Ratio model, through comparisons with mortality data of ten developed countries. The numerical examples show that the first proposed model maintains a comparable forecast ability with the existing methods. In contrast, the second proposed model outperforms the first model as well as the existing models in terms of forecast accuracy.

## 1. Introduction

There have been tremendous developments in the area of mortality modelling and forecasting over the last three decades. These include the pioneering mortality model proposed by [25], well-known as the Lee–Carter model. It rapidly gained credit and popularity, given its simplicity and ability to capture most variations in mortality patterns evolved over time. Several modifications and extensions of the Lee–Carter model have been put forward, see, for instance, Lee and Miller [26], Booth et al. [2], Renshaw and Haberman [34] and Currie et al [4]. It is worth noting that Hyndman and Ullah [19] further extend the Lee–Carter model to a functional data framework, which includes non-parametric smoothing techniques, functional principal component decomposition and times series analysis to achieve the task of mortality modelling and forecasting. Although the models as mentioned earlier posed a great success in history, the single factor designs limit their capacity of mortality modelling and forecasting on solely one population. It seems improper to prepare a mortality forecast for an individual population in isolation from one another if they are closely linked together. For example, due to biological and behavioural reasons, male mortality rates have consistently been higher than female mortality rates, see Kalben [22]. However, if male mortality improvements are faster than female ones, but two genders are projected independently, the model may forecast male mortality rates lower than and eventually diverge further from female mortality rates. As such, it is always a significant challenge in human mortality modelling that the model can take multiple populations as well as their heterogeneity simultaneously into account. Several mortality models for multiple populations have been proposed in the literature over the last decade, see, for instance, Delwarde et al. [10] and Dowd et al. [11]. In more desirable cases, the model can further ensure that the forecasts for multiple related populations maintain certain structural relationships based on the extensive theoretical considerations and historical observations. A ‘coherent’ or ‘non-divergent’ model is one of the most well-suited tools in mortality modelling given the fact that the mortality of populations that are geographically close or otherwise related is driven by a common set of factors such as socio-economic, environmental and biological conditions and differences are unlikely to increase in the long run. Such coherent forecast models are also documented in the literature, see, for example, the earliest augmented common factor (ACF) model proposed by Li and Lee [29], which is an extension of the Lee–Carter model with an additional common factor to capture both short-term divergence and long-term coherence among related populations. Variants and extensions of the ACF model have been subsequently developed, such as Li [27], Li et al. [28] and Chen and Millossovich [6]. Some others like the Age-Period-Cohort (APC) model proposed by Cairns et al. [5], incorporate a mean-reverting stochastic process for two related populations and allow for different trends in mortality improvement rates in the short-run but parallel improvements in the long-run. The Product-Ratio model developed by Hyndman et al. [17], which models the product and ratio functions of the age-specific mortality rates of different populations individually through a functional principal component decomposition, achieves coherent mortality forecasts by constraining the forecast ratio function via a stationary time series model to appropriate constants. Shang [35] and Wu and Wang [37] use multilevel functional principal component analysis of aggregated and population-specific data to extract the common trend and population-specific residual trend among populations. The forecast of population-specific residual trend is restricted to be a stationary time process to achieve convergence in the long run. Some other developments in this field include Jarner and Kryger [21], Hatzopoulos and Haberman [15] and Wan and Bertschi [36]. Also, see Danesi et al. [9] and Enchev et al. [12] for reviews and comparisons.

In this article, we propose two new models for mortality modelling and forecasting with the theoretical framework of multivariate functional principal component analysis techniques introduced by Chiou et al. [8] and Happ and Greven [14]. The main objectives of the multivariate functional principal component analysis are to carry out an eigendecomposition with all populations grouped together and model multiple sets of functional curves that may be correlated among others, which allows us to construct two new models on top of these ideas. The first proposed model is to treat the groups of population mortality rates within a large population equally and model them with similarities and correlations across ages and periods altogether for forecasting. The second proposed model is a novel method for coherent mortality modelling and forecasting that captures the common trend and the population-specific trend of groups of mortality patterns and produces forecasts of different populations that do not diverge and present convergence in the long run. It incorporates both overall information from the population as a whole and specific information from the subpopulations deviated from the overall information for mortality modelling and forecasting. The two proposed models are estimated using the weighted functional principal component algorithm with geometrically decaying weighting scheme [18], which assigns more weights to the most recent data than those in the distant past. This extension can produce more realistic forecasts and achieve improved forecast accuracies than the original proposal of the multivariate functional principal component analysis techniques when it comes to forecasting.

More will be discussed in detail in the article, and the rest of this article is organised as follows. In Section 2, we give a review of the theoretical background about univariate and multivariate functional principal component analyses. In Section 3, we describe the general frameworks of two proposed multivariate functional principal component analysis models for mortality modelling and forecasting. We then illustrate the models by applying them to the sex-specific mortality rates for Japan with comparisons to two analogous functional data paradigms − the independent FPCA model and the Product-Ratio model proposed by Hyndman and Ullah [19] and Hyndman et al. [17], in terms of the systematic differences and forecasting performances using sex-specific mortality data of ten developed countries in Section 4. We lastly conclude this article with discussions and remarks in Section 5.

## 2. Theoretical background of FPCA

Functional principal component analysis (FPCA) is the core technique applied primarily in this article. It is a statistical method for analysing the variation of a set of functional curves in a dataset then reducing them from infinite dimensions to finite dimensions in the principal component representations of variation [31]. It can also be regarded as a functional extension of the multivariate PCA method, allowing the data dimension to increase from finite space to infinite space [33]. After the Karhunen–Loève theorem in expansions of a stochastic process proposed by Karhunen [23] and Loève [30], the theoretical developments of FPCA can be divided into two main fields: the linear operator and the covariance operator perspectives, see, for example, Besse [1], Ramsay and Silverman [32], Yao et al. [38], Hall et al. [13] and Bosq [3]. To get the readers well equipped with the necessary concepts in this article, we firstly give a brief review of univariate FPCA then move on to discuss the algorithm of performing multivariate FPCA directly from the results of univariate FPCA.

### 2.1. Univariate FPCA (UFPCA)

Let Y(x) be a continuous and mean square integrable ( $L^{2}$-continuous) stochastic process on a domain X with a mean function μ(x)=E(Y(x)) and a covariance function $K(x,x^{\prime })=Cov(Y(x),Y(x^{\prime }))$ for all x∈X. Assuming that there exists a covariance operator $\Gamma :L^{2}(X)\to L^{2}(X)$ for any function $f\in L^{2}(X)$, we have

$$\left(\Gamma f\right)(x)=\int_{X}K(x,x^{\prime })f(x^{\prime })\;dx^{\prime },\;\forall \;x\in X.$$

With the defined covariance operator Γ, we can perform a spectral analysis of the covariance function $K(x,x^{\prime })$, such that

$$\left(\Gamma \phi \right)(x)=\int_{X}K(x,x^{\prime })\phi (x^{\prime })\;dx^{\prime }=\lambda \phi (x),$$

to obtain a set of orthonormal basis eigenfunctions $\{\phi_{n}(x){\}}_{n=1}^{\infty }$ and a corresponding set of eigenvalues $\{\lambda_{n}{\}}_{n=1}^{\infty }$, where $\lambda_{1}\geq \lambda_{2}\geq \cdots \geq 0$, representing the amount of variability in Y(x) explained by the $\{\phi_{n}(x){\}}_{n=1}^{\infty }$. Y(x) can now be represented as an infinite linear combination of the orthonormal functions by the Karhunen–Loève theorem, that is

$$Y(x)=\mu \left(x\right)+\sum_{n=1}^{\infty }{\text{β}}_{n}\phi_{n}(x),\;\forall \;x\in X,$$

where ${\text{β}}_{n}$ is the principal component score with

$${\text{β}}_{n}=\int_{X}(Y(x)-\mu (x))\phi_{n}(x)\;dx.$$

The principal component scores $\{{\text{β}}_{n}{\}}_{n=1}^{\infty }$ are uncorrelated random variables with mean zero and variance $\{\lambda_{n}{\}}_{n=1}^{\infty }$. ${\text{β}}_{n}$ serves as the weight and the projection of the centred stochastic process (Y(x)−μ(x)) in the direction of the *n*-th eigenfunction $\phi_{n}(x)$ in the Karhunen–Loève representation of Y(x). In practice, only the first few eigenfunctions are needed to represent the most important features of Y(x), we can therefore truncate the Karhunen–Loève expansion at the first *N*-dimensional terms to obtain an approximation of Y(x), i.e.

(1) $$Y(x)\approx \mu \left(x\right)+\sum_{n=1}^{N}{\text{β}}_{n}\phi_{n}(x),\;\forall \;x\in X,$$

and thus reduce the infinite dimension of functional data into finite dimensions in principal direction of variation which is often used for data analysis, e.g. for regression or for clustering [31].

### 2.2. Algorithm of performing multivariate FPCA (MFPCA) from the results of UFPCA

We now consider multivariate functional data and provide a natural path performing multivariate functional principal component analysis from the results of univariate functional principal component analysis using a simple algorithm introduced by Happ and Greven [14]. The main idea of the algorithm is derived from the theoretical framework of multivariate FPCA considering the covariance operator point of view, and its mathematical details can be found in the supplementary material of this article.

Given a random sample consisting of p≥2 sets of subpopulation functions $Y^{\left(1\right)}(x),\ldots ,Y^{\left(p\right)}(x)$ which are from the same population and have the variances on the same scale^1^ on a domain X for all x∈X, the MFPCA estimation algorithm comprises the following four steps:

1. Perform a univariate functional principal component analysis for each element $Y^{\left(i\right)}(x)$ consisting of the observed curves $\{Y_{t}^{\left(i\right)}(x){\}}_{t=1}^{T}$ with a subscript *t*, for t=1,…,T, as each observation unit.^2^ This gives us a set of estimated principal component scores $\{{\hat{\text{β}}}_{t,n}^{\left(i\right)}{\}}_{n=1}^{N}$ and estimated eigenfunctions $\{{\hat{\phi }}_{n}^{\left(i\right)}(x){\}}_{n=1}^{N}$ with the first suitably chosen *N*-dimensional approximations to each $Y^{\left(i\right)}(x)$.
2. Combine all the estimated principal component scores into a single large matrix Ξ where
$$\Xi =\left(\begin{array}{ccccccc} {\hat{\text{β}}}_{1,1}^{\left(1\right)} & \ldots & {\hat{\text{β}}}_{1,N}^{\left(1\right)} & \ldots & {\hat{\text{β}}}_{1,1}^{\left(p\right)} & \ldots & {\hat{\text{β}}}_{1,N}^{\left(p\right)}\\ \vdots & \ddots & \vdots & \ddots & \vdots & \ddots & \vdots \\ {\hat{\text{β}}}_{T,1}^{\left(1\right)} & \ldots & {\hat{\text{β}}}_{T,N}^{\left(1\right)} & \ldots & {\hat{\text{β}}}_{1,N}^{\left(p\right)} & \ldots & {\hat{\text{β}}}_{T,N}^{\left(p\right)} \end{array}\right)\in R^{T\times pN}$$
  and estimate the joint covariance matrix $\hat{Z}$ = $\frac{1}{N-1}\Xi^{T}\Xi$.
3. Perform a matrix eigenanalysis for $\hat{Z}$ to obtain a set of estimated orthonormal eigenvectors $\{{\hat{c}}_{n}{\}}_{n=1}^{N}$ and a set of corresponding eigenvalues $\{{\hat{\nu }}_{n}{\}}_{n=1}^{N}$ of $\hat{Z}$.
4. Calculate the estimated multivariate eigenfunctions and the estimated multivariate principal component scores according to their *i*-th elements:
$${\hat{\psi }}_{n}^{\left(i\right)}(x)=\sum_{m=1}^{N}[{\hat{c}}_{n}{]}_{m}^{\left(i\right)}{\hat{\phi }}_{m}^{\left(i\right)}(x),\;\forall \;x\in X,$$
  and
$${\hat{\rho }}_{t,n}=\sum_{i=1}^{p}\sum_{m=1}^{N}[{\hat{c}}_{n}{]}_{m}^{\left(i\right)}{\hat{\text{β}}}_{t,m}^{\left(i\right)}.$$

The empirical truncated multivariate Karhunen–Loève representation with the first *N*-dimensional approximations to $Y_{t}^{\left(i\right)}(x)$ is

$${\hat{Y}}_{t}^{\left(i\right)}(x)={\hat{\mu }}^{\left(i\right)}\left(x\right)+\sum_{n=1}^{N}{\hat{\rho }}_{t,n}{\hat{\psi }}_{n}^{\left(i\right)}(x),\;\forall \;x\in X,$$

where ${\hat{\mu }}^{\left(i\right)}\left(x\right)=\frac{1}{T}\sum_{t=1}^{T}Y_{t}^{\left(i\right)}(x)$, and the estimated multivariate principal component score ${\hat{\rho }}_{t,n}$ gives the individual weight of each observation unit *t* for its corresponding estimated multivariate eigenfunction ${\hat{\psi }}_{n}^{\left(i\right)}(x)$.

## 3. Methodology

### 3.1. Weighted MFPCA model for multipopulation mortality rates modelling and forecasting

In this section, we firstly introduce our new model, namely weighted MFPCA (wMFPCA) model for forecasting mortality rates of several subpopulations within a large population simultaneously.

Let $Y_{t}^{\left(i\right)}(x)$ denote the log of the observed mortality rates of the *i*-th subpopulation for age *x* in year *t*. We assume there is an underlying $L^{2}$-continuous and mean square integrable function $f_{t}^{\left(i\right)}(x)$ that we are observing with error and at discrete points of *x*. Our discrete observations are $\left\{x_{j},Y_{t}^{\left(i\right)}(x_{j})\right\}$, for i=1,…,p,t=1,…,T,j=1,…,J, such that

$$Y_{t}^{\left(i\right)}(x_{j})=f_{t}^{\left(i\right)}(x_{j})+\sigma_{t}^{\left(i\right)}(x_{j})e_{t,j}^{\left(i\right)},$$

where $\{e_{t,j}^{\left(i\right)}{\}}_{t,j=1}^{T,J}$ are i.i.d. standard normal random variables and $\sigma_{t}^{\left(i\right)}(x_{j})$ allows the amount of noise to vary with age *x*.

In demographic modelling, it is often the case that more recent experience has greater relevance on the future behaviour than those data from the distant past. In view of this, we comprise a weighted functional component algorithm for the MFPCA model, allowing the forecasting results of the model to be based more on the recent data.

Let ${\hat{f}}_{t}^{\left(i\right)}(x)$ be a smoothed function estimated from the observation $Y_{t}^{\left(i\right)}(x_{j})$, and $w_{t}=\kappa (1-\kappa {)}^{T-t}$ be a geometrically decaying weight with 0<κ<1. The overall mean function $\mu^{\left(i\right)}(x)$ of $Y_{t}^{\left(i\right)}(x)$ is estimated by the weighted average

$${\hat{\mu }}^{\left(i\right)}(x)=\sum_{t=1}^{T}w_{t}{\hat{f}}_{t}^{\left(i\right)}(x).$$

The mean-centred functional data is denoted as ${\hat{f}}_{t}^{*(i)}(x)={\hat{f}}_{t}^{\left(i\right)}(x)-\mu^{\left(i\right)}(x)$. We discretise ${\hat{f}}_{t}^{*(i)}(x)$ as a *T* by *J* matrix ${\hat{F}}^{(i)*}$, then multiply ${\hat{F}}^{(i)*}$ by W, where $W=diag(w_{1},\ldots ,w_{T})$, such that ${\hat{F}}^{\left(i\right)}=W{\hat{F}}^{(i)*}$. We then follow the algorithm of estimating MFPCA introduced in the previous section to calculate the weighted principal component scores and the weighted multivariate eigenfunctions using the functional form of ${\hat{F}}^{\left(i\right)}$ to obtain ${\hat{F}}_{t}^{\left(i\right)}(x)=\sum_{n=1}^{N}{\hat{\rho }}_{t,n}{\hat{\psi }}_{n}^{\left(i\right)}(x)$ up to the first *N*-dimensional approximations. We lastly combine the estimated weighted average with the estimated weighted multivariate eigenfunctions and the estimated multivariate weighted principal component scores to obtain the weighted MFPCA model for mortality modelling and forecasting of the *i*-th subpopulation with the first *N*-dimensional approximations, i.e.

$${\hat{Y}}_{t}^{\left(i\right)}(x)={\hat{\mu }}^{\left(i\right)}\left(x\right)+\sum_{n=1}^{N}{\hat{\rho }}_{t,n}{\hat{\psi }}_{n}^{\left(i\right)}(x)+{\hat{\sigma }}_{t}^{\left(i\right)}(x){\hat{e}}_{t}^{\left(i\right)}.$$

#### 3.1.1. Out-of-sample forecasts and prediction intervals of the wMFPCA model

Forecasts can be obtained by forecasting the weighted principal component scores $\{{\hat{\rho }}_{t,n}{\}}_{n=1}^{N}$ using time series models independently. There is no need to consider the vector autoregression (VAR) model for forecasting the weighted principal component scores as they are not correlated. $\{{\hat{\rho }}_{t,n}{\}}_{n=1}^{N}$ can be extrapolated using possibly non-stationary autoregressive integrated moving average (ARIMA) model and we can select the order of the ARIMA model based on the Akaike information criterion (AIC) or the Bayesian information criterion (BIC).

Let ${\hat{\rho }}_{t+h,n}$ denote the *h*-step ahead forecast of ${\hat{\rho }}_{t,n}$, then the *h*-step ahead out-of-sample forecast of ${\hat{Y}}_{t}^{\left(i\right)}(x)$ is

$${\hat{Y}}_{t+h}^{\left(i\right)}(x)={\hat{\mu }}^{\left(i\right)}\left(x\right)+\sum_{n=1}^{N}{\hat{\rho }}_{t+h,n}{\hat{\psi }}_{n}^{\left(i\right)}(x).$$

We can also obtain the forecasting variance of the model by adding up the variances of all terms together given the fact that the components of the wMFPCA model are uncorrelated, such that

$$Var(Y_{t+h}^{\left(i\right)}(x))={\hat{\tau }}_{{\hat{\mu }}^{\left(i\right)}}^{2}(x)+\sum_{n=1}^{N}{\hat{\nu }}_{t+h,n}({\hat{\psi }}_{n}^{\left(i\right)}(x){)}^{2}+({\hat{\sigma }}_{t+h}^{\left(i\right)}(x){)}^{2},$$

where ${\hat{\tau }}_{{\hat{\mu }}^{\left(i\right)}}^{2}(x)$ is the variance of the smoothed estimates of the mean function derived from the smoothing method applied, ${\hat{\nu }}_{t+h,n}$ is the estimated variance of ${\hat{\rho }}_{t+h,n}$ that can be obtained from the time series method used, and the estimated variance of forecast error $({\hat{\sigma }}_{t+h}^{\left(i\right)}(x){)}^{2}$ is calculated by taking the average of observational variance from the historical data.

With the normality assumption on the model error and the known $Var(Y_{t+h}^{\left(i\right)}(x))$, a 100(1−α)% prediction interval for ${\hat{Y}}_{t+h}^{\left(i\right)}(x)$ can be calculated as ${\hat{Y}}_{t+h}^{\left(i\right)}(x)\pm z_{\alpha }\sqrt{Var(Y_{t+h}^{\left(i\right)}(x))}$, where $z_{\alpha }$ is the (1−α/2) quantile of the standard normal distribution.

### 3.2. Coherent wMFPCA model for multipopulation mortality rates modelling and forecasting

We now introduce the idea of the coherent wMFPCA model, in the sense that the long-term forecasts of several subpopulations within a large population will be non-divergent.

Let $Y_{t}^{\left(i\right)}(x)$ be the log of the observed mortality rates of the *i*-th subpopulation for age *x* in year *t*, $\{e_{t}^{\left(i\right)}{\}}_{t=1}^{T}$ are the i.i.d. standard normal random variables, and $\sigma_{t}^{\left(i\right)}(x)$ allows the amount of noise varying with age *x*. The coherent wMFPCA model has the following form:

$$Y_{t}^{\left(i\right)}(x)=f_{t}^{\left(i\right)}(x)+\sigma_{t}^{\left(i\right)}(x)e_{t}^{\left(i\right)},$$

where

$$f_{t}^{\left(i\right)}(x)=\mu (x)+\eta^{\left(i\right)}(x)+G_{t}(x)+Z_{t}^{\left(i\right)}(x).$$

$f_{t}^{\left(i\right)}(x)$ is the smoothed mortality function of the *i*-th subpopulation for age *x* in year *t*, μ(x) is the average of total mortality function, $\eta^{\left(i\right)}(x)$ is the mean of the *i*-th subpopulation specific deviation function from the averaged total mortality function, $G_{t}(x)$ is the common trend across all populations, and $Z_{t}^{\left(i\right)}(x)$ is the *i*-th subpopulation-specific deviation trend.

In such a model, μ(x) and $\eta^{\left(i\right)}(x)$ are unknown fixed functions, while $G_{t}(x)$ and $Z_{t}^{\left(i\right)}(x)$ are assumed to be independent zero mean stochastic processes to ensure identifiability [35], such that $G_{t}(x)$ and $Z_{t}^{\left(i\right)}(x)$ can then be decomposed by the (multivariate) Karhunen–Loève representation as

$$\begin{array}{rl} & G_{t}(x)=\sum_{k=1}^{\infty }{\text{β}}_{t,k}\phi_{k}(x),\\ & Z_{t}^{\left(i\right)}(x)=\sum_{l=1}^{\infty }\gamma_{t,l}\varphi_{l}^{\left(i\right)}(x), \end{array}$$

where $\{{\text{β}}_{t,k}{\}}_{k=1}^{\infty }$ and $\{\phi_{k}(x){\}}_{k=1}^{\infty }$ are the corresponding principal component scores and the eigenfunctions of $G_{t}(x)$ while $\{\gamma_{t,l}{\}}_{l=1}^{\infty }$ and $\{\varphi_{l}^{\left(i\right)}(x){\}}_{l=1}^{\infty }$ are the corresponding multivariate principal component scores and the multivariate eigenfunctions of $Z_{t}^{\left(i\right)}(x)$. It follows that $\{{\text{β}}_{t,k}{\}}_{k=1}^{\infty }$ is uncorrelated with $\{\gamma_{t,l}{\}}_{l=1}^{\infty }$. Following these expansions, the model can be expressed as

$$f_{t}^{\left(i\right)}(x)=\mu (x)+\eta^{\left(i\right)}(x)+\sum_{k=1}^{\infty }{\text{β}}_{t,k}\phi_{k}(x)+\sum_{l=1}^{\infty }\gamma_{t,l}\varphi_{l}^{\left(i\right)}(x).$$

#### 3.2.1. Estimation of the coherent wMFPCA model

We carry on the same weighted functional component algorithm applied in the wMFPCA model for the coherent wMFPCA model. The components of the coherent wMFPCA model can be obtained using the estimation procedures below in practice:

1. Obtain the total mortality function among all subpopulations smoothed mortality functions, ${\hat{g}}_{t}(x)=\frac{1}{p}\sum_{i=1}^{p}{\hat{f}}_{t}^{\left(i\right)}(x)$, then calculate the weighted mean function of the total mortality function, $\hat{\mu }(x)=\sum_{t=1}^{T}w_{t}{\hat{g}}_{t}(x)$, where $w_{t}=\kappa (1-\kappa {)}^{T-t}$ is a geometrically decaying weight with 0<κ<1.
2. Calculate the centred functional data ${\hat{g}}_{t}^{*}(x)={\hat{g}}_{t}(x)-\hat{\mu }(x)$, then discretise ${\hat{g}}_{t}(x)$ as a *T* by *J* matrix $G^{*}$, then multiply $G^{*}$ by W, where $W=diag(w_{1},\ldots ,w_{T})$, such that $\hat{G}=WG^{*}$.
3. Perform univariate FPCA on the functional form of $\hat{G}$ to get ${\hat{G}}_{t}(x)=\sum_{k=1}^{K}{\hat{\text{β}}}_{t,k}{\hat{\phi }}_{k}(x)$ up to the first *K*-dimensional approximations. Let ${\tilde{g}}_{t}(x)=\hat{\mu }(x)+\sum_{k=1}^{K}{\hat{\text{β}}}_{t,k}{\hat{\phi }}_{k}(x)$ be the estimated weighted total mortality function.
4. Calculate the deviation of the *i*-th subpopulation specific mortality function from the estimated weighted total mortality function, ${\hat{d}}_{t}^{\left(i\right)}(x)={\hat{f}}_{t}^{\left(i\right)}(x)-{\tilde{g}}_{t}(x)$, then calculate the weighted mean of the *i*-th subpopulation specific deviation function, ${\hat{\eta }}^{\left(i\right)}(x)=\sum_{t=1}^{T}w_{t}{\hat{d}}_{t}^{\left(i\right)}(x)$.
5. Obtain the demeaned functional data ${\hat{z}}_{t}^{(i)*}(x)={\hat{d}}_{t}^{\left(i\right)}(x)-{\hat{\eta }}^{\left(i\right)}(x)$, then discretise ${\hat{z}}_{t}^{(i)*}(x)$ as a *T* by *J* matrix ${\hat{Z}}^{(i)*}$, then multiply ${\hat{Z}}^{(i)*}$ by W, where $W=diag(w_{1},\ldots ,w_{T})$, to have ${\hat{Z}}^{\left(i\right)}=W{\hat{Z}}^{(i)*}$.
6. Perform multivariate FPCA on the functional form of ${\hat{Z}}^{\left(i\right)}$ to obtain ${\hat{Z}}_{t}^{\left(i\right)}(x)=\sum_{l=1}^{L}{\hat{\gamma }}_{t,l}{\hat{\varphi }}_{l}^{\left(i\right)}(x)$ up to the first *L*-dimensional approximations.

With all the estimated components obtained above, we can represent the coherent wMFPCA model for mortality modelling and forecasting of the *i*-th subpopulation, i.e.

$${\hat{Y}}_{t}^{\left(i\right)}(x)=\hat{\mu }(x)+{\hat{\eta }}^{\left(i\right)}(x)+{\hat{G}}_{t}(x)+{\hat{Z}}_{t}^{\left(i\right)}(x)+{\hat{\sigma }}_{t}^{\left(i\right)}(x){\hat{e}}_{t}^{\left(i\right)},$$

or the full representation of the coherent wMFPCA model with the first *K*-dimensional approximations to the common trend and the first *L*-dimensional approximations to the *i*-th subpopulation deviation trend, such that

$${\hat{Y}}_{t}^{\left(i\right)}(x)={\hat{\mu }}^{\left(i\right)}(x)+\sum_{k=1}^{K}{\hat{\text{β}}}_{t,k}{\hat{\phi }}_{k}(x)+\sum_{l=1}^{L}{\hat{\gamma }}_{t,l}{\hat{\varphi }}_{l}^{\left(i\right)}(x)+{\hat{\sigma }}_{t}^{\left(i\right)}(x){\hat{e}}_{t}^{\left(i\right)},$$

where ${\hat{\mu }}^{\left(i\right)}(x)=\hat{\mu }(x)+{\hat{\eta }}^{\left(i\right)}(x)$ is the mean function of the *i*-th subpopulation.

#### 3.2.2. Out-of-sample forecasts and prediction intervals of the coherent wMFPCA model

The *h*-step ahead out-of-sample forecast of $Y_{t}^{\left(i\right)}(x)$ can be represented as

$${\hat{Y}}_{t+h}^{\left(i\right)}(x)={\hat{\mu }}^{\left(i\right)}(x)+\sum_{k=1}^{K}{\hat{\text{β}}}_{t+h,k}{\hat{\phi }}_{k}(x)+\sum_{l=1}^{L}{\hat{\gamma }}_{t+h,l}{\hat{\varphi }}_{l}^{\left(i\right)}(x),$$

where ${\hat{\text{β}}}_{t+h,k}$ and ${\hat{\gamma }}_{t+h,l}$ are the *h*-step ahead forecasts of the weighted principal component scores of the common trend and the *i*-th subpopulation specific deviation trend. ${\hat{\text{β}}}_{t+h,k}$ can be obtained using a univariate time series forecasting method, such as ARIMA model. To ensure the predictions of the subpopulations are coherent in the long term, the forecasts of all subpopulation deviation trends need to be restricted to be convergent and a stationary process, such that $\lim_{h\to \infty }\sum_{l=1}^{L}({\hat{\gamma }}_{t+h,l}{\hat{\varphi }}_{l}^{\left(i\right)}(x)-{\hat{\gamma }}_{t+h,l}{\hat{\varphi }}_{l}^{\left(j\right)}(x))=0$. ${\hat{\gamma }}_{t+h,k}$ can hence be achieved using possibly any stationary autoregressive moving average (ARMA) process or autoregressive fractionally integrated moving average (ARFIMA) process. The order of the aforementioned time series models can be decided based on the Akaike information criterion (AIC) or the Bayesian information criterion (BIC).

Given the way that the coherent wMFPCA model has been constructed, each component is independent of the other components. Therefore, the forecast variance can be expressed by the sum of component variances, i.e.

$$Var(Y_{t+h}^{\left(i\right)}(x))={\hat{\tau }}_{{\hat{\mu }}^{\left(i\right)}}^{2}(x)+\sum_{k=1}^{K}{\hat{u}}_{t+h,k}({\hat{\phi }}_{k}(x){)}^{2}+\sum_{l=1}^{L}{\hat{\omega }}_{t+h,l}({\hat{\varphi }}_{l}^{\left(i\right)}(x){)}^{2}+({\hat{\sigma }}_{t+h}^{\left(i\right)}(x){)}^{2},$$

where ${\hat{\tau }}_{{\hat{\mu }}^{\left(i\right)}}^{2}(x)$ is the variance of the smoothed estimates of the mean function derived from the smoothing method used, ${\hat{u}}_{t+h,k}$ and ${\hat{\omega }}_{t+h,l}$ are the variances of ${\hat{\text{β}}}_{t+h,k}$ and ${\hat{\gamma }}_{t+h,l}$ that can be obtained from the time series methods applied, and the forecast error $({\hat{\sigma }}_{t+h}^{\left(i\right)}(x){)}^{2}$ is the average of the observational variance estimated from the historical data.

With the normality assumption on the model error and the known $Var(Y_{t+h}^{\left(i\right)}(x))$, a 100(1−α)% prediction interval for ${\hat{Y}}_{t+h}^{\left(i\right)}(x)$ can be calculated as ${\hat{Y}}_{t+h}^{\left(i\right)}(x)\pm z_{\alpha }\sqrt{Var(Y_{t+h}^{\left(i\right)}(x))}$, where $z_{\alpha }$ is the (1−α/2) quantile of the standard normal distribution.

Note that the weights $\{w_{t}{\}}_{t=1}^{T}$ are controlled by the tuning parameter *κ* in the geometrically decaying weighting approach embedded in the two proposed models. The larger *κ* is, the faster the weights for the historical observations are decaying over time geometrically. In practice, the tuning parameter *κ* can be determined by minimising the average root mean square error (RMSE) of all populations defined as

(2) $$RMSE=\frac{1}{p}\sum_{i=1}^{p}\sqrt{\frac{1}{J}\sum_{j=1}^{J}(Y_{t+h}^{\left(i\right)}(x_{j})-{\hat{Y}}_{t+h}^{\left(i\right)}(x_{j}){)}^{2}}.$$

The value of the parameter *κ* can alternatively be specified as a prior, if there is a strong prior knowledge of how past data should be weighted [37].

For selecting the number of principal components in the two proposed models, we use a cumulative percentage of total variation method. We denote *N* as a generic notation of the number of principal components chosen, and *N* is determined by

$$N=\underset{N:N\geq 1}{argmin}(\frac{\sum_{n=1}^{N}{\hat{\lambda }}_{n}}{\sum_{n=1}^{\infty }{\hat{\lambda }}_{n}}\geq P),$$

where ${\hat{\lambda }}_{n}$ is the corresponding estimated eigenvalue of the principal components analysis, and *P* = 0.9 is set to be the minimum acceptance level as suggested by Chiou [7].

## 4. Applications

In this section, we illustrate the two proposed models − the wMFPCA model and the coherent wMFPCA model using sex-specific mortality data. We first present and plot the observed mortality dataset, then demonstrate the usefulness of these two models by forecasting of the sex-specific mortality rates of Japan. We show the forecasting results for males and females compared with the observed data. We further exhibit the ability of non-diverging long-term forecasts of the proposed coherent wMFPCA model and finally assess the forecasting accuracy of the two proposed models in comparison to the Product-Ratio model and the independent FPCA model using the sex-specific mortality data of ten different developed countries.

### 4.1. Sex-specific mortality data of Japan

**Figure 1.**
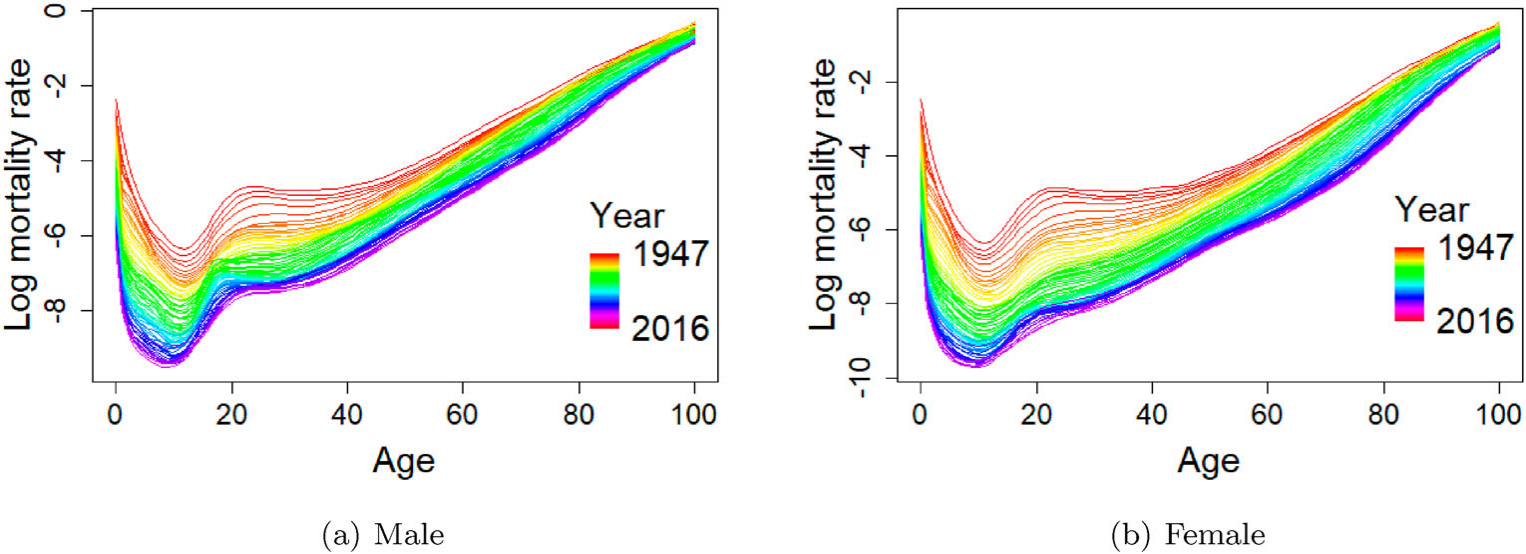
Smoothed log mortality rates for males and females from the year 1947 to the year 2016 in Japan, viewed as functional data curves with time-ordering indicated by the colours of the rainbow from red to violet. (a) Male (b) Female.

The sex-specific mortality data of Japan are available for the year 1947 to the year 2016 from the Human Mortality Database [16]. The database consists of central death rates by gender and single calendar year of age up to 110 years old. We restrict the data at the maximum age of 100 to avoid problems associated with erratic rates above age 100. The observed mortality rates curves are smoothed using penalised regression splines with a partial monotonic constraint so that each mortality curve is increasing above age 65 monotonically [19]. Figure 1 presents the sets of observed male and female mortality data as a batch of smoothed curves (functional observation), respectively in a rainbow plot with time-ordering indicated by the colours of the rainbow, from red to violet. Figure 1 shows that there are steady declines in male and female mortality rates at most ages over the examined period. The mortality curve patterns for male and female are reasonably similar, while for male, the mortality rates are generally higher than the mortality rates of female, particularly at around age 20. Despite the higher male mortality rates in comparison with female's, the mortality gap between male's and female's gets narrower over time at older ages.

### 4.2. Sex-specific mortality modelling and forecasting by the wMFPCA model

**Figure 2.**
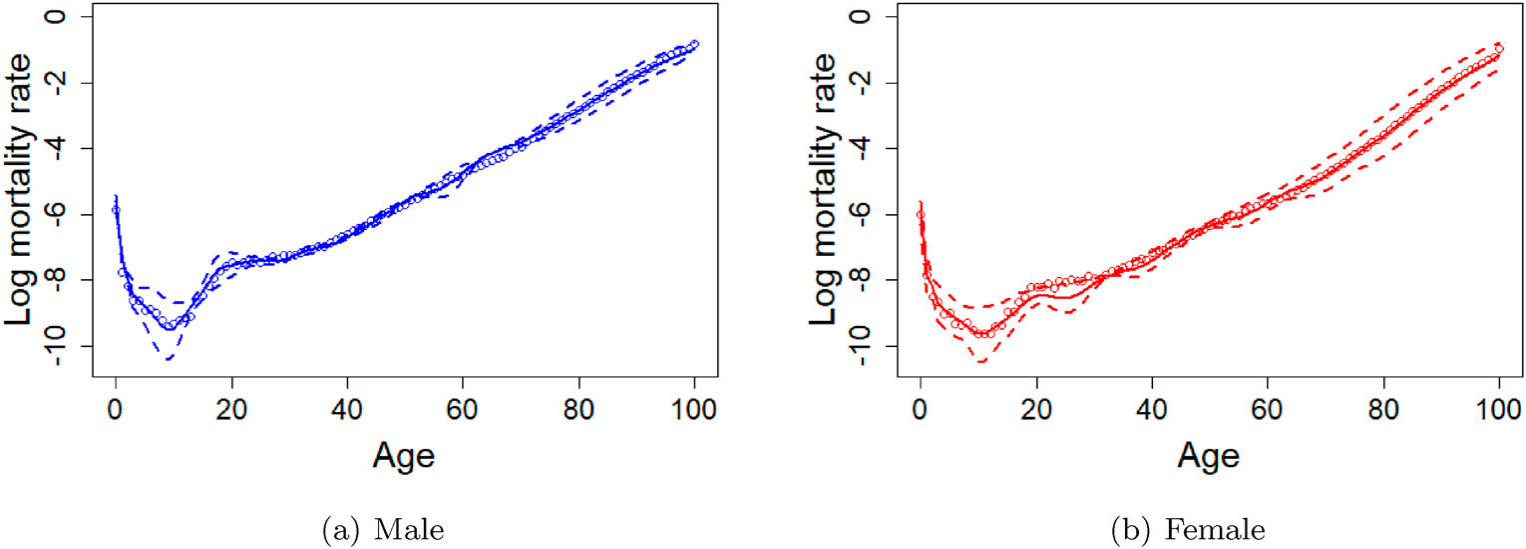
Predicted mortality curves of male (with RMSE = 0.2006) and for female (with RMSE = 0.2406) from age 0 to age 100 with the 95% prediction intervals using the wMFPCA model for the year 2016 based on the observations from the year 1947 to the year 1996 in Japan. Circles are the true log mortality rates, solid lines are the predictions, and dashed lines are the 95% prediction intervals. (a) Male (b) Female.

In the demonstration of the first proposed weighted MFPCA model, we aim to make 20-years-ahead out-of-sample forecasts for male and female mortality rates in Japan. We first split the dataset with the observed mortality rates from the year 1947 to the year 1996 and a test dataset with the remaining observed mortality data from the year 1997 to the year 2016. We decide the value of the weight parameter over the interval 0<κ<1 that can minimise the average root mean square error (RMSE) stated in Equation (2) of male and female mortality data based on a rolling window approach; see Section 4.5 for the details. The mean functions for male and female and their functional principal components are estimated as discussed in the previous section. The analysis shows that the first three functional principal components for male and female explain 97.2%, 2.3% and 0.2%, respectively, which account for more than 99% in total of the variations in the sample data and are above the minimum 90% acceptance level. We, therefore, select the first three estimated principal components for approximations and demonstrations. For each score of the corresponding functional principal component shared by male and female, we forecast it independently by a univariate ARIMA time series using the R package ‘ forecast’ [20]. The order of ARIMA models is chosen based on the Akaike information criterion (AIC). The figures and the descriptions of the estimated functional principal components and their corresponding scores for male and female using the wMFPCA model can be found in the supplementary materials of this article. Figure 2 shows the 20-years-ahead forecasting results of mortality curves of male (with RMSE = 0.2006) and female (with RMSE = 0.2406) from age 0 to age 100 for the year 2016 by the wMFPCA model based on the in-sample data from the year 1947 to the year 1996 in Japan.

### 4.3. Sex-specific mortality modelling and forecasting by the coherent wMFPCA model

**Figure 3.**
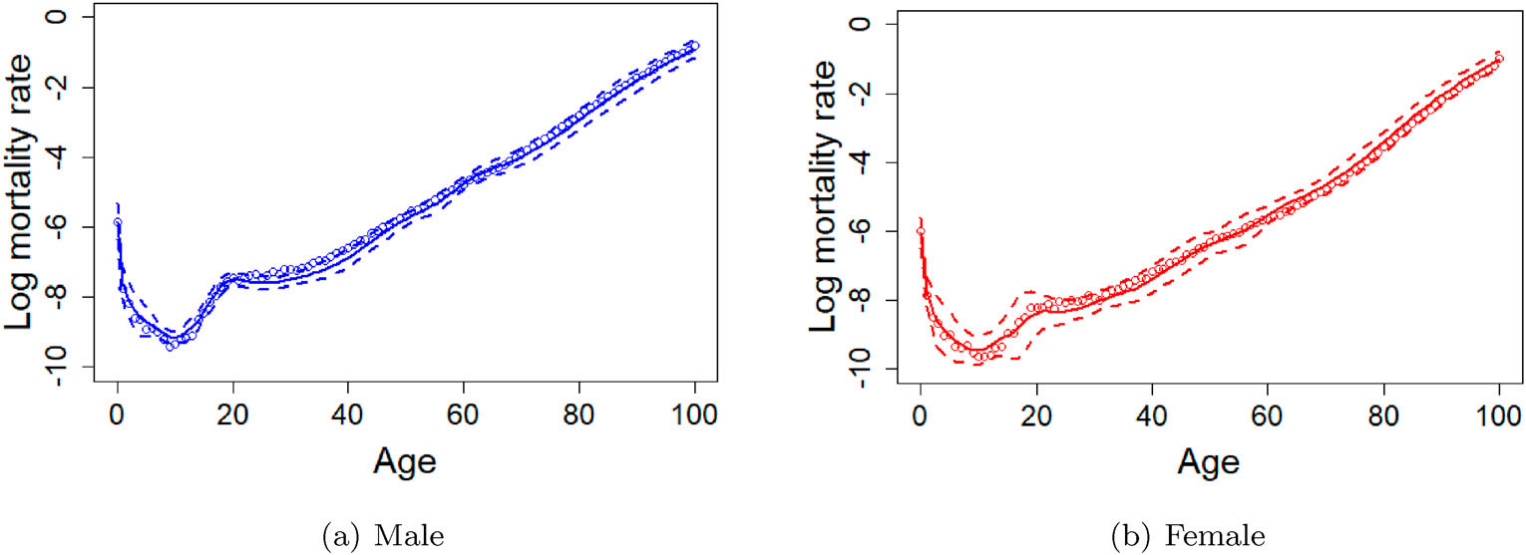
Predicted mortality curves of male (with RMSE = 0.1821) and female (with RMSE = 0.1460) from age 0 to age 100 with the 95% prediction intervals using the coherent wMFPCA model for the year 2016 based on the observations from the year 1947 to the year 1996 in Japan. Circles are the true log mortality rates, solid lines are the predictions, and dashed lines are the 95% prediction intervals. (a) Male (b) Female.

The presentation of the coherent wMFPCA model forecasting follows the same strategies of how we split the dataset for in- and out-of-sample data and choosing the weight parameter for the coherent wMFPCA model as we have done for the wMFPCA model in the previous section. The figures and the descriptions of the estimated functional principal components and their corresponding scores for male and female using the coherent wMFPCA model can also be found in the supplementary materials of this article. Figure 3 shows the 20-years-ahead forecasting results of mortality curves of male (with RMSE = 0.1821) and female (with RMSE = 0.1460) from age 0 to age 100 for the year 2016 using the coherent wMFPCA model based on the in-sample data from the year 1947 to the year 1996 in Japan.

### 4.4. Forecast pattern of life expectancy curves by the coherent and the non-coherent forecasting methods

In this section, we move on to examine and compare forecast patterns with mortality sex ratios and life expectancy by the forecasts of the two proposed models − the wMFPCA model and the coherent wMFPCA model with four different approaches − the independent FPCA model [18], the unweighted MFPCA model, the Product-Ratio model [17] and the weighted multilevel FPCA model proposed by Wu and Wang [37].

The independent FPCA model is a univariate FPCA method for forecasting two subpopulations independently without considerations of any potential correlation of them. The unweighted MFPCA model uses the theoretical framework proposed by Happ and Greven [14] combined with the same extrapolation method as the wMFPCA model but without any time weighting. The forecast results of the independent FPCA model, the unweighted MFPCA model and the wMFPCA model are based on a non-stationary time series model on their estimated principal component scores, leading to forecast results of two or more subpopulations divergent to different directions in the long run. They are thus regarded as a non-coherent forecasting approach. Meanwhile, the Product-Ratio model begins with an idea of obtaining the product and ratio function of all subpopulations by assuming all subpopulations have equal variance. In the log scale, the product function can be treated as the sum of all sub populations, whereas the ratio function can be treated as the differences among subpopulations. The predictions can be obtained by firstly applying the independent FPCA model to forecast the future realisations of the product and ratio functions separately, then transforming the forecasts of the product and ratio functions back to the original subpopulations functions. The convergent forecasts are achieved by using stationary time series methods, namely the ARMA model or the ARFIMA model, on the ratio function, which implicitly implies that the differences among subpopulations will be convergent to zero in the long term. It is, therefore, viewed as an example of a coherent forecasting approach. In a similar vein, the weighted multilevel FPCA model and the proposed coherent wMFPCA model both restrict the stationary properties on the deviation functions of each subpopulation from the overall mean to accomplish the coherent forecasting with no need to assume all the subpopulations have the same variances.

**Figure 4.**
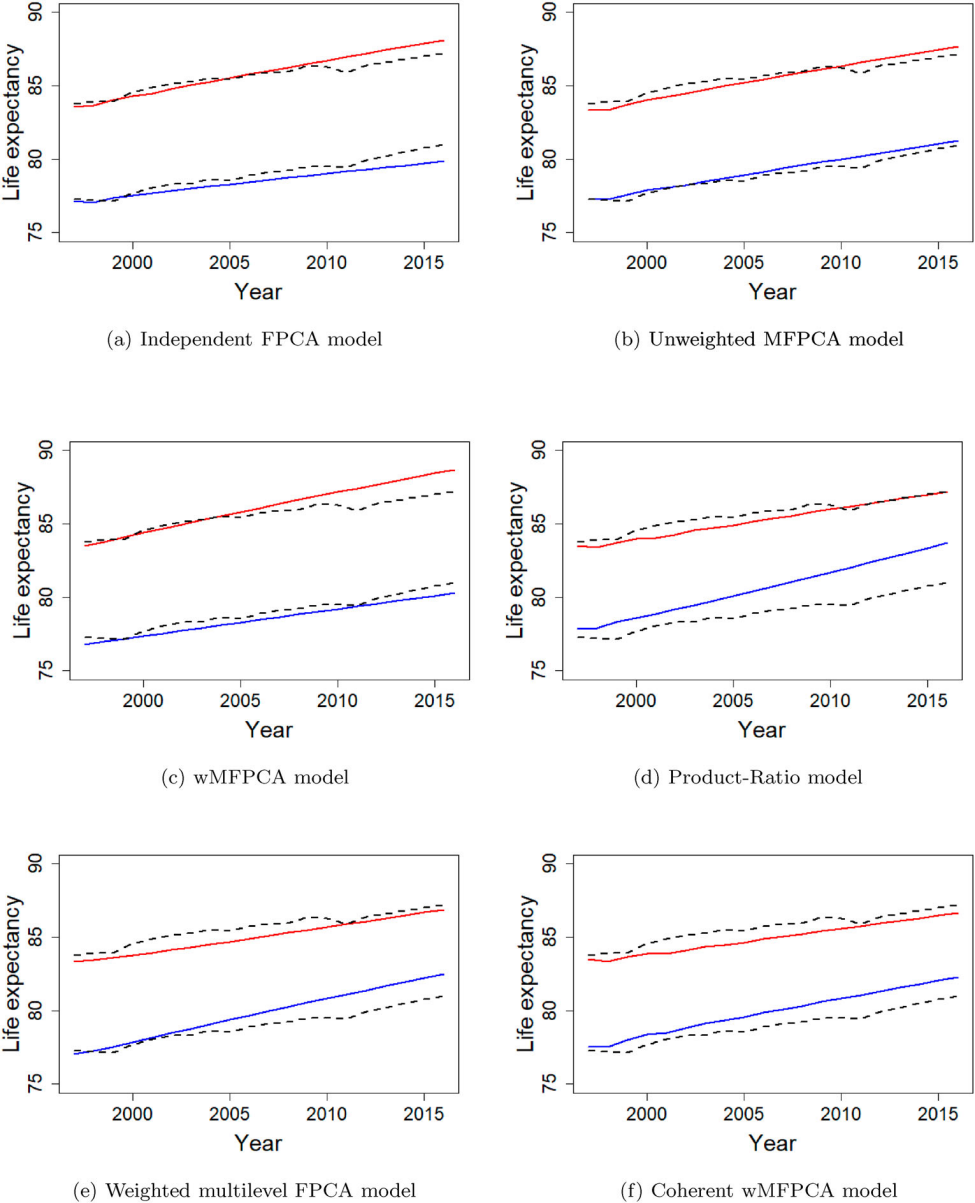
20-year life expectancy predicted curves for male and female in Japan using the independent FPCA model, the unweighted MFPCA model, the wMFPCA model, the Product-Ratio model, the weighted multilevel FPCA model and the coherent wMFPCA model. Blue sold line is used for male and red sold line is used for female. Dotted lines are the observed life expectancy for males and females. (a) Independent FPCA model, (b) Unweighted MFPCA model, (c) wMFPCA model, (d) Product-Ratio model, (e) Weighted multilevel FPCA model, (f) Coherent wMFPCA model.

To deliver the concept of coherent forecasting more concretely, we plot the life expectancy curves obtained from the observed male and female mortality rates from the year 1997 to the year 2016 alongside the 20-years-ahead forecasts of the life expectancy curves from the year 1997 to the year 2016 by the non-coherent forecast methods − the independent FPCA model, the unweighted MFPCA model and the wMFPCA model and the coherent forecast methods − the Product-Ratio model, the weighted multilevel FPCA model and the coherent wMFPCA model using the observed mortality rates from the year 1947 to the year 1996 in Figure 4. We can see that the convergent forecastings by the coherent models fit well with the actual biological characteristics trends in Figure 4, where the differences in males and females life expectancy converge to a certain level gradually and slowly, instead of diverging into different directions like the forecast results of the non-coherent forecast methods. The demonstration shows the importance of coherent modelling when there exist common biological characteristics among several subpopulations. See the supplementary material of this article for more details on the comparison of the historical and predicted patterns of mortality sex ratios (Male/Female) by the coherent and the non-coherent forecasting methods.

### 4.5. Forecast accuracy evaluation with comparisons to other existing methods

We now evaluate and compare the forecast accuracy of our two proposed models − the wMPFCA model and the coherent wMFPCA model with the four different approaches − the independent FPCA model, the unweighted MFPCA model, the Product-Ratio model and the weighted multilevel FPCA model demonstrated in the previous section.

In order to have a comprehensive investigation of the forecast accuracy of our two proposed models, we consider ten other developed countries for which data are also available in the Human Mortality Database. We restrict data periods of all selected countries commencing in the year 1950 up to the year 2010 for a unified purpose. We examine and quantify the forecasting performance of our models by a rolling window analysis, which is frequently used for assessing the consistency of a model's forecasting ability by rolling a fixed-size prediction interval (window) throughout the observed sample [39]. We hold the sample data from the initial year up to the year *t* as holdout samples. We produce the forecast for the *t* + *h* year where *h* is the forecast horizon, then determine the forecasts errors by comparing the forecast result with the actual out-of-sample data. We increase one rolling window (1 year ahead) in year *t* + 1 to make the same procedure again for the year *t* + *h* + 1 until the rolling window analysis covers all available data.

We include four different forecast horizons (h=5,10,15 and 20) with ten sets of rolling window to exam the short-term, the mid-term and the long-term forecast abilities of the two proposed models. We use the root mean square error (RMSE) to measure the standard deviation of the average square prediction error regardless of sign. In our experiments, we define the RMSE as follows:

$${RMSE}_{c}^{\left(i\right)}(h)=\sqrt{\frac{1}{10\times 101}\sum_{w=0}^{9}\sum_{j=1}^{101}(Y_{t+w+h}^{\left(i\right)}(x_{j})-{\hat{Y}}_{t+w+h}^{\left(i\right)}(x_{j}){)}^{2}},$$

where *c* is the selected country, *w* is the rolling window set, *i* is the subpopulation for male (i= M) and for female (i= F) and *j* is the age group including from age 0 to age 100 in our experiment.

Based on the average RMSE results of ten sets of rolling window analysis across ten countries in four different forecast horizons presented in Table 1, the proposed wMFPCA model shows to be more desirable for forecasting female mortality in Australia and Belgium, while the independent FPCA model is particularly good for the long-term forecasting of female mortality in Italy and Spain. We can see that the forecasting performances between the weighted multilevel FPCA model and the proposed coherent wMFPCA model are comparable. The weighted multilevel FPCA model performs the best in terms of having the lowest average female forecast errors in the short run and obtaining relatively smaller male forecast errors in the long run than the other models. Meanwhile, the proposed coherent wMFPCA method shows to be more capable of capturing rapid changes in male mortality in the short-term forecasting and keeping female mortality forecast errors relatively lower than the other models in the long-term forecasting across different periods and age groups among the most considered countries. The forecast performances of the unweighted MFPCA model and the Product-Ratio model are reasonably similar with no particular outstanding area than the others.

**Table 1.** Forecast accuracy of mortality for male and female using the independent FPCA model, the unweighted MFPCA model, the wMFPCA model, the Product-Ratio model, the weighted multilevel FPCA model, and the coherent wMPFCA model is measured by the average RMSEs of ten sets of rolling windows analysis.

	Independent FPCA model			Unweighted MFPCA model			wMFPCA model			Product-Ratio model			Weighted multilevel FPCA model			Coherent wMFPCA model		
Country	M	F	$\frac{M+F}{2}$	M	F	$\frac{M+F}{2}$	M	F	$\frac{M+F}{2}$	M	F	$\frac{M+F}{2}$	M	F	$\frac{M+F}{2}$	M	F	$\frac{M+F}{2}$
*h* = 5																		
Australia	0.1605	0.1436	0.1520	0.1623	0.1449	0.1536	0.1573	**0.1429**	0.1501	0.1622	0.1429	0.1525	0.1513	0.1511	0.1512	**0.1485**	0.1474	**0.1479**
Belgium	0.1747	0.1885	0.1816	0.1755	0.1880	0.1818	0.1701	**0.1814**	0.1757	0.1687	0.1909	0.1798	**0.1545**	0.1960	0.1753	0.1560	0.1866	**0.1713**
Canada	0.1337	0.1072	0.1204	0.1305	0.1075	0.1190	0.1308	0.1107	0.1208	0.1328	**0.1025**	0.1176	0.1223	0.1060	0.1142	**0.1122**	0.1045	**0.1083**
France	0.1297	0.1068	0.1182	0.1313	0.1054	0.1183	0.1374	**0.1066**	0.1220	0.1277	0.1193	0.1235	0.1096	0.1147	0.1122	**0.1037**	0.1184	**0.1110**
Italy	0.1988	0.1791	0.1889	0.1963	0.1661	0.1812	0.2028	0.1723	0.1875	0.2123	0.1741	0.1932	0.1797	**0.1433**	0.1615	**0.1679**	0.1487	**0.1583**
Japan	0.1127	0.1103	0.1115	0.1140	0.1124	0.1132	**0.1058**	0.1233	0.1146	0.1197	0.1003	0.1100	0.1366	0.0908	0.1137	0.1175	**0.0835**	**0.1005**
Netherlands	0.1362	0.1583	0.1472	0.1445	0.1579	0.1512	0.1416	0.1546	0.1481	0.1481	0.1474	0.1478	0.1282	**0.1437**	**0.1359**	**0.1274**	0.1444	0.1359
Spain	0.2140	0.1805	0.1973	0.2133	0.2011	0.2072	**0.2046**	0.1951	0.1999	0.2444	0.1681	0.2062	0.2375	**0.1459**	**0.1917**	0.2375	0.1841	0.2108
U.K	**0.1019**	0.1153	0.1086	0.1057	0.1061	0.1059	0.1066	0.1081	0.1074	0.1225	0.0967	0.1096	0.1131	0.0923	0.1027	0.1085	**0.0913**	**0.0999**
U.S.A	0.0895	0.0802	0.0848	0.0933	0.0859	0.0896	**0.0891**	0.0902	0.0897	0.0933	0.0720	0.0826	0.1080	**0.0562**	0.0821	0.0910	0.0616	**0.0763**
Average	0.1452	0.1370	0.1411	0.1467	0.1375	0.1421	0.1446	0.1385	0.1416	0.1532	0.1314	0.1423	0.1441	**0.1240**	0.1340	**0.1370**	0.1270	**0.1320**
*h* = 10																		
Australia	0.2178	0.1614	0.1896	0.2151	0.1567	0.1859	0.2129	0.1555	0.1842	0.2134	**0.1535**	0.1834	0.1977	0.1598	0.1787	**0.1888**	0.1554	**0.1721**
Belgium	0.2208	0.2073	0.2141	0.2248	0.2043	0.2146	0.2058	**0.1957**	0.2008	0.1989	0.2193	0.2091	**0.1710**	0.2270	0.1990	0.1751	0.2138	**0.1945**
Canada	0.1935	0.1342	0.1639	0.1944	0.1367	0.1656	0.1983	0.1442	0.1712	0.1877	0.1222	0.1549	0.1646	**0.1199**	0.1422	**0.1577**	0.1241	**0.1409**
France	0.2260	0.1690	0.1975	0.2180	0.1686	0.1933	0.2195	**0.1661**	0.1928	0.2049	0.1956	0.2003	**0.1520**	0.1725	**0.1623**	0.1577	0.1782	0.1679
Italy	0.2703	0.2512	0.2608	0.2607	0.2421	0.2514	0.2694	0.2550	0.2622	0.2804	0.2398	0.2601	0.2430	**0.2089**	**0.2260**	**0.2376**	0.2163	0.2269
Japan	0.1705	0.1964	0.1834	0.1605	0.1962	0.1783	**0.1636**	0.2303	0.1969	0.1918	0.1524	0.1721	0.2618	0.1645	0.2132	0.1904	**0.1169**	**0.1537**
Netherlands	0.1814	0.1929	0.1872	0.2008	0.1908	0.1958	0.1920	0.1899	0.1910	0.1883	0.1625	0.1754	0.1598	**0.1578**	**0.1588**	**0.1596**	0.1594	0.1595
Spain	0.3829	0.2689	0.3259	0.3444	0.3104	0.3274	0.3448	0.3109	0.3279	0.3517	0.2863	0.3190	0.3594	**0.2604**	**0.3099**	**0.3287**	0.3006	0.3147
U.K	**0.1600**	0.1769	0.1684	0.1665	0.1636	0.1650	0.1684	0.1716	0.1700	0.1992	0.1353	0.1672	0.1776	**0.1163**	0.1470	0.1670	0.1177	**0.1424**
U.S.A	0.1418	0.1222	0.1320	0.1459	0.1334	0.1396	0.1421	0.1426	0.1424	**0.1384**	0.1097	0.1240	0.1485	**0.0822**	**0.1153**	0.1535	0.0884	0.1210
Average	0.2165	0.1880	0.2023	0.2131	0.1903	0.2017	0.2117	0.1962	0.2039	0.2155	0.1777	0.1966	0.2035	**0.1669**	0.1852	**0.1916**	0.1671	**0.1793**
*h* = 15																		
Australia	0.2800	0.2056	0.2428	0.2766	0.2117	0.2442	0.2806	**0.2004**	0.2405	0.2711	0.2073	0.2392	0.2493	0.2088	0.2291	**0.2487**	0.2018	**0.2252**
Belgium	0.2798	0.2365	0.2581	0.2917	0.2397	0.2657	0.2656	**0.2306**	0.2481	0.2452	0.2605	0.2528	**0.2126**	0.2703	0.2415	0.2240	0.2551	**0.2395**
Canada	0.2514	0.1716	0.2115	0.2578	0.1743	0.2160	0.2614	0.1861	0.2238	0.2194	0.1512	0.1853	**0.1860**	0.1489	0.1675	0.1877	**0.1458**	**0.1668**
France	0.3087	**0.2069**	0.2578	0.3000	0.2199	0.2599	0.2906	0.2105	0.2505	0.2511	0.2724	0.2618	**0.1776**	0.2361	**0.2069**	0.2043	0.2497	0.2270
Italy	0.3101	0.2570	0.2836	0.2985	0.2461	0.2723	0.3060	0.2621	0.2841	0.2805	0.2669	0.2737	**0.2284**	**0.2386**	**0.2335**	0.2445	0.2453	0.2449
Japan	0.2333	0.2841	0.2587	0.2091	0.2870	0.2480	**0.2221**	0.3323	0.2772	0.2609	0.2158	0.2384	0.3704	0.2335	0.3019	0.2570	**0.1605**	**0.2087**
Netherlands	0.2298	0.2339	0.2319	0.2670	0.2361	0.2515	0.2496	0.2320	0.2408	0.2222	0.1883	0.2053	**0.1860**	0.1868	**0.1864**	0.1924	**0.1856**	0.1890
Spain	0.4820	**0.3051**	0.3936	0.4015	0.3673	0.3844	0.4097	0.3714	0.3906	0.3753	0.3593	0.3673	0.3901	0.3290	0.3595	**0.3191**	0.3564	**0.3377**
U.K	**0.2277**	0.2440	0.2358	0.2299	0.2269	0.2284	0.2323	0.2402	0.2363	0.2736	0.1754	0.2245	0.2342	**0.1420**	**0.1881**	0.2298	0.1508	0.1903
U.S.A	0.1617	0.1601	0.1609	0.1709	0.1691	0.1700	0.1665	0.1828	0.1747	0.1417	0.1291	0.1354	**0.1407**	**0.1023**	**0.1215**	0.1512	0.1129	0.1320
Average	0.2764	0.2305	0.2535	0.2703	0.2378	0.2541	0.2684	0.2448	0.2566	0.2541	0.2226	0.2384	0.2375	0.2096	0.2236	**0.2259**	**0.2064**	**0.2161**
*h* = 20																		
Australia	0.3657	0.2337	0.2997	0.3612	0.2433	0.3022	0.3595	**0.2295**	0.2945	0.3311	0.2508	0.2909	**0.3047**	0.2545	**0.2796**	0.3162	0.2526	0.2844
Belgium	0.3537	0.2589	0.3063	0.3665	0.2626	0.3146	0.3411	**0.2493**	0.2952	0.2976	0.2999	0.2988	**0.2494**	0.3101	**0.2798**	0.2751	0.3024	0.2888
Canada	0.3126	0.1985	0.2556	0.3210	0.2013	0.2612	0.3243	0.2144	0.2693	0.2564	0.1716	0.2140	**0.2185**	0.1743	**0.1964**	0.2246	**0.1707**	0.1976
France	0.3984	**0.2572**	0.3278	0.3922	0.2623	0.3273	0.3818	0.2585	0.3202	0.3068	0.3357	0.3213	**0.2314**	0.3040	**0.2677**	0.2702	0.3215	0.2958
Italy	0.3917	**0.2613**	0.3265	0.3779	0.2486	0.3132	0.3820	0.2681	0.3251	0.3094	0.3036	0.3065	**0.2428**	0.2833	**0.2631**	0.2803	0.2937	0.2870
Japan	0.3021	0.3798	0.3410	0.2692	0.3808	0.3250	**0.2917**	0.4374	0.3645	0.3298	0.2853	0.3076	0.4742	0.3224	0.3983	0.3256	**0.2195**	**0.2725**
Netherlands	0.3159	0.2521	0.2840	0.3394	0.2584	0.2989	0.3424	0.2538	0.2981	0.2980	0.2186	0.2583	**0.2486**	0.2340	**0.2413**	0.2675	**0.2186**	0.2431
Spain	0.6624	**0.3784**	0.5204	0.5497	0.4627	0.5062	0.5636	0.4694	0.5165	0.4735	0.4688	0.4712	0.4727	0.4357	0.4542	**0.3838**	0.4357	**0.4097**
U.K	0.2966	0.2982	0.2974	0.2939	0.2753	0.2846	0.2981	0.2952	0.2967	0.3310	0.2157	0.2733	0.2867	**0.1711**	**0.2289**	**0.2841**	0.1823	0.2332
U.S.A	0.1993	0.1948	0.1970	0.2068	0.2062	0.2065	0.1984	0.2173	0.2079	**0.1668**	0.1464	0.1566	0.1730	**0.1295**	**0.1513**	0.1922	0.1473	0.1697
Average	0.3598	0.2713	0.3156	0.3478	0.2802	0.3140	0.3483	0.2893	0.3188	0.3100	0.2696	0.2898	0.2902	0.2619	0.2761	**0.2820**	**0.2544**	**0.2682**

Theminimal forecast errors amongmodels in different forecast horizons are highlighted in bold.

When we focus on the forecast horizon size up to twenty years for long-term forecasting, we can see that the coherent models maintain relatively less forecast bias among two sexes than the non-coherent models. For instance, the independent FPCA model, the unweighted MFPCA model and the proposed wMFPCA model both produce comparatively large forecasting errors for male mortality and give small forecast inaccuracies for female mortality. In contrast, the coherent models can keep the same level of forecast errors for both genders. With assumed joint biological characteristics among the two genders that we discussed in the previous section, the mortality pattern among two sexes is supposed to get similar in the long run, and the convergent designed forecast model is therefore needed. In particular, the coherent wMFPCA model shows to be more suitable and accurate than the Product-Ratio model and the weighted multilevel FPCA model as it produces the smallest overall forecast errors and bias for both genders and across all the different forecast horizons and the tested countries in our study.

The main finding in this section is that in the two-sex case, the accuracy of the male forecast is considerably improved by the coherent models at the small expense of accuracy in female mortality forecasts. By adopting the coherent forecasting, the forecast accuracy among all subpopulations is homogeneous as it incorporates additional information into the forecast for a single subpopulation. The additional information acts as a frame of reference limiting to the probability of a subpopulation forecast which may continue a diverging trend from other related subpopulations directions.

## 5. Discussion and conclusion remarks

With the theoretical framework of multivariate functional principal component analysis motivated by Chiou et al. [8] and Happ and Greven [14], in this article, we have proposed two new models that aim to model and forecast for a group of mortality rates, taking advantages of commonalities in their historical experience and age patterns. The first one, namely as wMFPCA model, is introduced to acknowledge differences in groups, age patterns and trends of several subpopulations to model together when subpopulations have somewhat sufficiently similar historical patterns. The coherent wMFPCA model is a novel extension of the wMFPCA model in a coherent direction. We design the coherent structure of the model to primarily fulfil the idea that when several subpopulation groups have similar socio-economic conditions or common biological characteristics and such these close connections are expected to continue and evolve in a non-diverging fashion in the distant future. The time weighting approaches on these two models lead us to expect the future patterns of mortality to follow more likely recent past observations and obliterate some parts of irrelevant distant past mortality movements in favour of forecast performances of the two proposed models.

We have demonstrated the two proposed models through forecasting for sex-specific mortality with the observed data from Japan. The wMFPCA model consists of the mean functions and the functional principal components of each subpopulation with corresponding scores shared by all subpopulations. We can obtain the forecasts of the wMFPCA model by extrapolating the shared principal component scores ahead with any non-stationary time series model, such as ARIMA model in the numerical examples. Coherence is another important issue that aims to be addressed in this article. The coherent wMFPCA model includes two primary components: the average components among all subpopulations and each subpopulation's component that deviated from the average component. The coherence is ensured by applying a stationary time series model for the forecasts of the deviation components corresponding PC scores. Under the stationary time series scheme, it guarantees that the extrapolated PC scores of the deviation components converge so that the deviation components also converge to their age-specific constants in the long run. As the long-term forecasts of all the subpopulation's deviation components converge to constants and all different subpopulations share the same average components in the proposed model, they gradually lose the ability to affect the mortality change, and their impacts on the mortality change are also equal in the long term. Therefore, the forecasted mortality differences among all subpopulations are constrained, leading to a similar constraint on the predicted life expectancy curves among all subpopulations in the meantime. The whole population's mortality change is eventually dominated by the long-term forecasts of the average components. This non-divergent forecastability of the proposed coherent wMFPCA model is confirmed by the forecasts of mortality sex ratios and life expectancy cures in the numerical examples of this article.

It is worth mentioning that Shang [35] and Wu and Wang [37] also proposed a similar approach using the multilevel functional principal component analysis framework for coherent mortality forecasting. The multilevel functional principal component structure relies on the set of the subpopulation-specified PC scores for different subpopulations but sharing the same set of eigenfunctions among all the subpopulations in the deviation components, which implies that the subpopulation-specified PC scores are not independent and hence some multivariate or vector autoregressive moving average models with stationary restriction are required to extrapolate all the correlated subpopulation-specified PC scores for coherent forecasting [37]. Moreover, estimating the subpopulation-specified PC scores using the multilevel functional principal component analysis for multilevel function data involves extra difficulties because the shared eigenfunctions in different levels are not necessarily mutually orthogonal. Some additional assumptions may thus be needed to estimate the subpopulation-specified PC scores and the shared eignfunctions in a probabilistic structure [37]. In contrast, the multivariate functional principal component framework that we adapted for the proposed coherent wMFPCA model provides a much more straightforward idea to achieve the same coherent forecasting task than the multilevel functional principal component approach. The proposed coherent wMFPCA model allows each subpopulation to have its own set of eigenfunctions but sharing the same set of PC scores among all the subpopulations. Given that the shared PC scores are uncorrelated, there is no need to consider multivariate or vector autoregressive models when we extrapolate the set of the shared PC scores for forecasting. Coherent forecasts can be achieved simply by extrapolating the set of PC scores in each dimension using some stationary time series models independently. The estimation of the shared PC scores is also straightforward with no extra assumptions imposed because the eigenfunctions in the multivariate FPCA framework are mutually orthogonal.

The usefulness of the two proposed models is illustrated through a series of forecast accuracy evaluations and comparisons with other existing methods. The first proposed wMFPCA model provides a very flexible framework for multipopulation mortality forecasting with comparable forecast accuracy as the independent FPCA model, the unweighted MFPCA model and the Product-Ratio model. The second proposed coherent wMFPCA model outperforms the Product-Ratio model in terms of forecast accuracy with no assumptions needed to place on the equal variance of all subpopulations. The proposed coherent wMFPCA model also shows to have a comparable overall short-term forecasting performance with the weighted multilevel FPCA model but outperforms it in the long-term forecasting and avoids the usage of multivariate time series models for forecasting. Although the numerical results show that the independent functional method gives relatively better forecast accuracy results for females in some developed countries than the other multipopulation and coherent models, these outcomes are something that we expected. The small variabilities and the good consistency of female mortality among some developed countries can contribute to the independent functional method with more superior forecast performances as it does not include the information from male mortality with high instabilities for female mortality forecasting. However, the coherence property becomes essential when it comes to human mortality modelling and forecasting as some multiple related populations always maintain certain structural relationships supported by the extensive theoretical considerations and historical observations, such as the non-diverging mortality patterns among males and females. The independent FPCA model may provide slightly better forecast accuracy for females in the short run, but the model may lead to the male mortality rates eventually diverged further from the female mortality rates in the long term as demonstrated in the numerical examples in Section 4.4. In contrast, the proposed coherent wMFPCA model maintains a comparable short-term forecast ability as the independent FPCA model and only trades off a relatively small amount of forecast accuracy for females in exchange for more sensible forecast results with less forecast error and bias in the two-sex mortality case in the long term, and this is the main justification that explains the importance and the advantage of the proposed coherent wMFPCA model over the independent functional method. This feature of the coherent wMFPCA model is also useful in some other specific practical applications, such as financial planning with several related stock prices, in a situation that we aim to maintain a balanced error margin amongst all subpopulations. This speciality is unique and has not been achieved by other non-coherent or single population models.

The main limitations of the two proposed models also attribute to the characteristics in which they belong to the classes of ‘non-parametric’ or ‘pure extrapolative’ methods. They can capture trends in the historical data well. At the same time, they lack the ability to incorporate more other related information, such as the change in medical technology, environment and social-economy for predictions. Another issue is the compatibility of the wMFPCA models. It requires a certain level of homogeneity among the observed functional time series curves across different time periods and among subpopulations for modelling and forecasting. The ability of the wMFPCA models may be affected if several completely irrelevant subpopulations are placed together in the wMFPCA models. Also, if the observed functional time series contains extreme realisations and cannot be regarded as approximately coming from the same stochastic process, this could result in unsatisfactory forecast accuracy. In this case, as suggested by Lee [24], one approach is to first partition the curves into different relatively homogenous groups and then apply FPCA. Another solution is to use a moving window approach to perform FPCA for the curves within a time window. As the window can contain a shorter time-span the mortality curves inside the window will have less variation and hence could be better treated as samples from the same stochastic process.

## Supplementary Material

Supplemental Material

## Notes

1 A standardisation may require for the p≥2 sets of functions if their variances are not on the same scale prior to the MFPCA estimation algorithm.

2 Note that here we notate *t* as each observation unit as *t* will be used to index the observed mortality curves across years on an age domain in the later mortality data application.

## References

[1] P. Besse, PCA stability and choice of dimensionality, Stat. Probab. Lett. 13 (1992), pp. 405–410.

[2] H. Booth, J. Maindonald, and L. Smith, Applying Lee-Carter under conditions of variable mortality decline, Popul. Stud. 56 (2002), pp. 325–336. 10.1080/00324720215935 12553330

[3] D. Bosq, *Linear Processes in Function Spaces: Theory and Applications*, Springer, New York, 2000.

[4] A.J.G. Cairns, D. Blake, and K. Dowd, Modelling and management of mortality risk: A review, Scand. Actuar. J. 2008 (2008), pp. 79–113.

[5] A.J.G. Cairns, D. Blake, K. Dowd, G.D. Coughlan, and M. Khalaf-Allah, Bayesian stochastic mortality modelling for two populations, ASTIN Bull. 41 (2011), pp. 29–59.

[6] R.Y. Chen and P. Millossovich, Sex-specific mortality forecasting for UK countries: A coherent approach, Eur. Actuar. J. 8 (2018), pp. 69–95. 29974029 10.1007/s13385-017-0164-0 PMC6004005

[7] J.M. Chiou, Dynamical functional prediction and classification, with application to traffic flow prediction, Ann. Appl. Stat. 6 (2012), pp. 1588–1614.

[8] J.M. Chiou, Y.F. Yang, and Y.T. Chen, Multivariate functional linear regression and prediction, J. Multi. Anal. 146 (2016), pp. 301–312.

[9] I.L. Danesi, S. Haberman, and P. Millossovich, Forecasting mortality in subpopulations using Lee-Carter type models: A comparison, Insur.: Math. Econ. 62 (2015), pp. 151–161.

[10] A. Delwarde, M. Denuit, M. Guillén, and A. Vidiella-i-Anguera, Application of the Poisson log-bilinear projection model to the G5 mortality experience, Belgian Actuar. Bull. 6 (2006), pp. 54–68.

[11] K. Dowd, A.J.G. Cairns, D. Blake, G.D. Coughlan, and M. Khalaf-Allah, A gravity model of mortality rates for two related populations, North Am. Actuar. J. 15 (2011), pp. 334–356.

[12] V. Enchev, T. Kleinow, and A.J.G. Cairns, Multi-population mortality models: fitting, forecasting and comparisons, Scand. Actuar. J. 2017 (2017), pp. 319–342.

[13] P. Hall, H.G. Müller, and J.L. Wang, Properties of principal component methods for functional and longitudinal data analysis, Ann. Stat. 34 (2006), pp. 1493–1517.

[14] C. Happ and S. Greven, Multivariate functional principal component analysis for data observed on different (dimensional) domains, J. Am. Stat. Assoc. 113 (2018), pp. 649–659.

[15] P. Hatzopoulos and S. Haberman, Common mortality modeling and coherent forecasts. an empirical analysis of worldwide mortality data, Insur.: Maths. Econ. 52 (2013), pp. 320–337.

[16] Human Mortality Database, University of California, Berkeley (USA), and Max Planck Institute for Demographic Research (Germany), 2020. Available at https://www.mortality.org/.

[17] R.J. Hyndman, H. Booth, and F. Yasmeen, Coherent mortality forecasting: the product-ratio method with functional time series models, Demography 50 (2013), pp. 261–283. 23055234 10.1007/s13524-012-0145-5

[18] R.J. Hyndman and H.L. Shang, Forecasting functional time series, J. Korean. Stat. Soc. 38 (2009), pp. 199–211.

[19] R.J. Hyndman and M.S. Ullah, Robust forecasting of mortality and fertility rates: A functional data approach, Comput. Stat. Data Anal. 51 (2007), pp. 4942–4956.

[20] R.J. Hyndman and Y. Khandakar, Automatic time series for forecasting: the forecast package for R, J. Stat. Softw. 27 (2008), pp. 1–22.

[21] S.F. Jarner and E.M. Kryger, Modelling adult mortality in small populations: The SAINT model, ASTIN Bull. 41 (2011), pp. 377–418.

[22] B.B. Kalben, Why men die younger: Causes of mortality differences by sex, North Am. Actuar. J. 4 (2000), pp. 83–111.

[23] K. Karhunen, Zur spektraltheorie stochastischer prozesse, Annales Academiae Scientiarum Fennicae 37 (1946), pp. 1–37.

[24] T.C.M. Lee, Discussion: forecasting functional time series, J. Korean. Stat. Soc. 38 (2009), pp. 217.

[25] R.D. Lee and L. Carter, Modeling and forecasting the time series of US mortality, J. Am. Stat. Assoc. 87 (1992), pp. 659–671.

[26] R.D. Lee and T. Miller, Evaluating the performance of the Lee-Carter method for forecasting mortality, Demography 38 (2001), pp. 537–549. 11723950 10.1353/dem.2001.0036

[27] J. Li, A Poisson common factor model for projecting mortality and life expectancy jointly for females and males, Popul. Stud. 67 (2013), pp. 111–126. 10.1080/00324728.2012.689316 22788919

[28] J. Li, L. Tickle, and N Parr, A multi-population evaluation of the Poisson common factor model for projecting mortality jointly for both sexes, J. Popul. Res. 33 (2016), pp. 333–360.

[29] N. Li and R.D. Lee, Coherent mortality forecasts for a group of populations: An extension of the Lee-Carter method, Demography 42 (2005), pp. 575–594. 16235614 10.1353/dem.2005.0021 PMC1356525

[30] M. Loève, Fonctions aléatoires à décomposition orthogonale exponentielle, La Revue Scientifique 84 (1946), pp. 159–162.

[31] J.O. Ramsay and B.W. Silverman, Applied functional data analysis: Methods and case studies, J. Am. Stat. Assoc. 100 (2005), pp. 577–590.

[32] J.O. Ramsay and B.W. Silverman, *Functional Data Analysis*, Springer, New York, 2005.

[33] J.O. Ramsay and C.J. Dalzell, Some tools for functional data analysis, J. R. Stat. Soc. Ser. B Statist. Methodol. 53 (1991), pp. 539–561.

[34] A. Renshaw and S. Haberman, A cohort-based extension to the Lee-Carter model for mortality reduction factors, Insur.: Math. Econ. 38 (2006), pp. 556–570.

[35] H.L. Shang, Mortality and life expectancy forecasting for a group of populations in developed countries: A multilevel functional data method, Ann. Appl. Stat. 10 (2016), pp. 1639–1672.

[36] C. Wan and L. Bertschi, Swiss coherent mortality model as a basis for developing longevity de-risking solutions for Swiss pension funds: A practical approach, Insur.: Maths. Econ. 63 (2015), pp. 66–75.

[37] R. Wu and B. Wang, Coherent mortality forecasting by the weighted multilevel functional principal component approach, J. Appl. Stat. 46 (2019), pp. 1774–1791.

[38] F. Yao, H.G. Müller, and J.L. Wang, Functional data analysis for sparse longitudinal data, J. Am. Stat. Assoc. 100 (2005), pp. 577–590.

[39] E. Zivot and J. Wang, *Modeling Financial Time Series with S-Plus ®*, Springer, New York, 2007.

