# Supplementary material for: Multipopulation mortality modelling and forecasting: the weighted multivariate functional principal component approaches
Source: J Appl Stat. 2022 Aug 3;50(15):3177–98. doi: 10.1080/02664763.2022.2104228 (PMC10631385; doi:10.1080/02664763.2022.2104228)
Supplement: Supplemental Material [file CJAS_A_2104228_SM7086.pdf]

SUPPLEMENTARY MATERIAL

Supplementary Material to "Multipopulation mortality modelling and forecasting: the weighted multivariate functional principal component approaches"

Ka Kin Lam and Bo Wang

School of Mathematics and Actuarial Science, University of Leicester, Leicester LE1 7RH, UK

ARTICLE HISTORY
Compiled August 7, 2022

1. Theoretical background of multivariate FPCA (MFPCA) from the covariance operator

We provide the mathematical details and illustrate the way to establish a direct link from the Karhunen–Loève representation of univariate functional data to the Karhunen–Loève representation of multivariate functional data.

We begin by considering a random sample which consists of $p \geq 2$ sets of functions $Y^{(1)}(x), \cdots, Y^{(p)}(x)$ on a domain $\mathcal{X}$ for all $x \in \mathcal{X}$. Combining all different sets of functions in a vector $\boldsymbol{Y}(x)$, we have

$$\boldsymbol{Y}(x) = (Y^{(1)}(x), \cdots, Y^{(p)}(x))^T \in \mathbb{R}^p$$

with a mean function

$$\boldsymbol{\mu}(x) = (\mathbb{E}(Y^{(1)}(x)), \cdots, \mathbb{E}(Y^{(p)}(x)))^T = (\mu^{(1)}(x), \cdots, \mu^{(p)}(x))^T,$$

and a covariance function

$$K_{ij}(x, x') = \text{Cov}(Y^{(i)}(x), Y^{(j)}(x')) = \mathbb{E}[(Y^{(i)}(x) - \mu^{(i)}(x))(Y^{(j)}(x') - \mu^{(j)}(x'))].$$

Let a set of functions $\boldsymbol{f} = (f^{(1)}, \cdots, f^{(p)})$ with an index for each $i = 1, \cdots, p$: $f^{(i)} \in L^2(\mathcal{X})$. Assuming that there exists a covariance operator $\Gamma : L^2(\mathcal{X}) \to L^2(\mathcal{X})$ for all $f \in L^2(\mathcal{X})$ with the i-th element of (Γf), we have

$$(\Gamma f)^{(i)}(x) = \sum_{j=1}^{p} \int_{\mathcal{X}} K_{ij}(x, x') f^{(j)}(x') dx', \quad \forall x \in \mathcal{X}.$$

With the defined covariance operator Γ and the similar structure in the univariate FPCA, we let $\big(\Gamma\psi\big)(x) = \nu\psi(x)$ by the spectral theorem on the covariance function

CONTACT Ka Kin Lam. Email: lamkakin@live.hk

$K_{ij}(x,x')$, where $\psi(x)$ is the orthonormal basis of eigenfunctions and ν is the corresponding eigenvalue. Then for all $i,j=1,\cdots,p$ and $x\in\mathcal{X}$, we have

$$(\Gamma\psi)^{(i)}(x)=\sum_{j=1}^{p}\int_{\mathcal{X}}K_{ij}(x,x')\psi^{(j)}(x')dx'=\nu\psi^{(i)}(x).$$

Without any loss of generality, we assume that each set of the functions $Y^{(1)}(x),\cdots,Y^{(p)}(x)$ has its finite univariate Karhunen–Loève representation up to the first N-dimensional approximations, i.e. $Y^{(i)}(x)=\mu^{(i)}(x)+\sum_{n=1}^{N}\beta_n^{(i)}\phi_n^{(i)}(x)$, and $K_{ij}(x,x')$ is a separable covariance function, i.e. $K_{ij}(x,x')=K_i(x,x')\cdot K_j(x,x')$. It holds for each $m,l=1,\cdots,N$ and $i,j=1,\cdots,p$ and for all $x\in\mathcal{X}$,

$$\begin{aligned}(\Gamma\psi)^{(i)}(x)&=\sum_{j=1}^{p}\sum_{m=1}^{N}\sum_{l=1}^{N}\int_{\mathcal{X}}\mathrm{Cov}(\beta_m^{(i)}\phi_m^{(i)}(x),\beta_l^{(j)}\phi_l^{(j)}(x'))\psi^{(j)}(x')dx'\\&=\sum_{j=1}^{p}\sum_{m=1}^{N}\sum_{l=1}^{N}\mathrm{Cov}(\beta_m^{(i)},\beta_l^{(j)})\phi_m^{(i)}(x)\int_{\mathcal{X}}\phi_l^{(j)}(x')\psi^{(j)}(x')dx'=\nu\psi^{(i)}(x).\end{aligned}\quad(1)$$

For simplicity of notation, we denote $\mathrm{Cov}(\beta_m^{(i)},\beta_l^{(j)})=Z_{ml}^{(ij)}$ and $\int_{\mathcal{X}}\phi_l^{(j)}(x')\psi^{(j)}(x')dx'=c_l^{(j)}$. Equation (1) can be rewritten as

$$\sum_{j=1}^{p}\sum_{m=1}^{N}\sum_{l=1}^{N}Z_{ml}^{(ij)}\phi_m^{(i)}(x)c_l^{(j)}=\nu\psi^{(i)}(x).\quad(2)$$

Following a similar technique of Zemyan [1] in solving Fredholm integral equations of the second kind with a separable covariance function, we can multiply and integrate an orthonormal basis eigenfunction $\phi_n^{(i)}(x)$, for $n=1,\cdots,N$, over the domain $\mathcal{X}$ for both sides of Equation (2),

$$\int_{\mathcal{X}}\phi_n^{(i)}(x)\cdot\sum_{j=1}^{p}\sum_{m=1}^{N}\sum_{l=1}^{N}Z_{ml}^{(ij)}\phi_m^{(i)}(x)c_l^{(j)}dx=\nu\int_{\mathcal{X}}\phi_n^{(i)}(x)\cdot\psi^{(i)}(x)dx.$$

Due to the orthonormality, $\int_{\mathcal{X}}\phi_n^{(i)}(x)\cdot\phi_m^{(i)}(x)dx=1$ if $m=n$, and $\int_{\mathcal{X}}\phi_n^{(i)}(x)\cdot\phi_m^{(i)}(x)dx=0$ if $m\neq n$ otherwise. Denoting $\int_{\mathcal{X}}\phi_n^{(i)}(x)\cdot\psi^{(i)}(x)dx=c_n^{(i)}$, it holds with the simplified notations

$$\sum_{j=1}^{p}\sum_{l=1}^{N}Z_{nl}^{(ij)}c_l^{(j)}=\nu c_n^{(i)}.\quad(3)$$

For a given value of ν, the solvability of this linear system correlates with the solvability of the integral equation. Since i,j and n,m,l are arbitrarily notated, Equation (3) can be represented in matrix form when we consider it as a whole, which is equivalent to an eigenequation, i.e.

$$\boldsymbol{Zc}=\nu\boldsymbol{c},\quad(4)$$

or

$$\begin{pmatrix} \boldsymbol{Z}^{(11)} & \dots & \boldsymbol{Z}^{(1p)} \\ \vdots & \ddots & \vdots \\ \boldsymbol{Z}^{(p1)} & \dots & \boldsymbol{Z}^{(pp)} \end{pmatrix} \begin{pmatrix} \boldsymbol{c}^{(1)} \\ \vdots \\ \boldsymbol{c}^{(p)} \end{pmatrix} = \nu \begin{pmatrix} \boldsymbol{c}^{(1)} \\ \vdots \\ \boldsymbol{c}^{(p)} \end{pmatrix}$$

with a positive semidefinite block matrix $\boldsymbol{Z}^{(ij)} \in \mathbb{R}^{N\times N}$ and an eigenvector $\boldsymbol{c}^{(i)} \in \mathbb{R}^N$ entries. With a matrix eigenanalysis performed on Equation (4), we can obtain a set of orthonormal eigenvectors $\{\boldsymbol{c_n}\}_{n=1}^N$ of $\boldsymbol{Z}$, corresponding a set of eigenvalues $\{\nu_n\}_{n=1}^N$, where $\nu_1 \geq \cdots \geq \nu_N \geq 0$.

Substituting the orthonormal eigenvector $\boldsymbol{c_n}$ and the corresponding eigenvalue ν_n into Equation (2), the eigenfunction $\psi_n^{(i)}(x)$ of Γ is given by their elements:

$$\psi_n^{(i)}(x) = \frac{1}{\nu_n} \sum_{j=1}^{p} \sum_{m=1}^{N} \sum_{l=1}^{N} Z_{ml}^{(ij)} [\boldsymbol{c_n}]_l^{(j)} \phi_m^{(i)}(x) = \sum_{m=1}^{N} [\boldsymbol{c_n}]_m^{(i)} \phi_m^{(i)}(x), \quad \forall x \in \mathcal{X},$$

where $[\boldsymbol{c_n}]^{(i)}$ denotes the i-th block of the orthonormal eigenvector $\boldsymbol{c_n}$ of $\boldsymbol{Z}$ corresponding to its eigenvalue ν_n. The truncated multivariate Karhunen–Loève expansions with the first N-dimensional approximations to $Y^{(i)}(x)$ can be written as

$$Y^{(i)}(x) = \mu^{(i)}(x) + \sum_{n=1}^{N} \rho_n \psi_n^{(i)}(x), \quad \forall x \in \mathcal{X}, \tag{5}$$

where ρ_n is the multivariate principal component score with

$$\begin{aligned} \rho_n &= \sum_{i=1}^{p} \int_{\mathcal{X}} (Y^{(i)}(x) - \mu^{(i)}(x)) \psi_n^{(i)}(x) dx \\ &= \sum_{i=1}^{p} \int_{\mathcal{X}} (Y^{(i)}(x) - \mu^{(i)}(x)) \sum_{m=1}^{N} [\boldsymbol{c_n}]_m^{(i)} \phi_m^{(i)}(x) \\ &= \sum_{i=1}^{p} \sum_{m=1}^{N} [\boldsymbol{c_n}]_m^{(i)} \int_{\mathcal{X}} (Y^{(i)}(x) - \mu^{(i)}(x)) \phi_m^{(i)}(x) = \sum_{i=1}^{p} \sum_{m=1}^{N} [\boldsymbol{c_n}]_m^{(i)} \beta_m^{(i)}, \end{aligned}$$

where $\beta_m^{(i)}$ is the m-th univariate principal component score of the i-th element. The mean and the covariance of ρ_n can be derived for all $i, j = 1, \cdots, p$ and $x \in \mathcal{X}$ as

$$\mathbb{E}(\rho_n) = \sum_{i=1}^{p} \int_{\mathcal{X}} \mathbb{E}(Y^{(i)}(x) - \mu^{(i)}(x)) \psi_n^{(i)}(x) dx = 0,$$

since $\mathbb{E}((Y^{(i)}(x) - \mu^{(i)}(x)) = 0$, and

$$\begin{aligned}
\text{Cov}(\rho_n, \rho_m) &= \mathbb{E}\Big(\sum_{i=1}^{p}\int_{\mathcal{X}}(Y^{(i)}(x) - \mu^{(i)}(x))\psi_n^{(i)}(x)dx \cdot \sum_{j=1}^{p}\int_{\mathcal{X}}(Y^{(j)}(x') - \mu^{(j)}(x'))\psi_m^{(j)}(x')dx'\Big) \\
&= \sum_{i=1}^{p}\int_{\mathcal{X}}\sum_{j=1}^{p}\int_{\mathcal{X}} K_{ij}(x, x')\psi_m^{(j)}(x')dx' \cdot \psi_n^{(i)}(x)dx \\
&= \sum_{i=1}^{p}\int_{\mathcal{X}} \nu_m\psi_m^{(i)}(x) \cdot \psi_n^{(i)}(x)dx \\
&= \nu_m \sum_{i=1}^{p}\int_{\mathcal{X}} \psi_m^{(i)}(x) \cdot \psi_n^{(i)}(x)dx.
\end{aligned} \tag{6}$$

Because of the orthonormality, $\int_{\mathcal{X}} \psi_m^{(i)}(x) \cdot \psi_n^{(i)}(x)dx = 1$ if $m = n$, and $\int_{\mathcal{X}} \psi_m^{(i)}(x) \cdot \psi_n^{(i)}(x)dx = 0$ if $m \neq n$ otherwise.

2. Application – Additional results

2.1. Sex-specific mortality modelling and forecasting by the wMFPCA model

2.1.1. Estimated functional principal components and their corresponding scores for male and female using the wMFPCA model

Figure 1 demonstrates the estimated mean functions $\hat{\mu}^{(i)}(x)$ and the first three estimated functional principal components $\{\hat{\psi}_n^{(i)}(x)\}$ for male ($i =$ M) and female ($i =$ F) using wMFPCA model for the sex-specific Japanese mortality data. Their corresponding scores of the PCs $\{\hat{\rho}_{t,n}\}_{n=1}^{3}$ with a 20-years-ahead out-of-sample forecast horizon and 95% prediction intervals are displayed in Figure 2. The functional principal components model different movements in mortality rates. The first functional principal components for male and female show a similar pattern, which both count for very high variation for young teenagers, then become level-off in middle-age and elderly. Although the corresponding scores for the first component show an increasing trend, we can interpret that there is a decreasing trend in mortality rates across our observed period given its corresponding first components for male and female are both negative. The second and the third principal components for male have relatively high negative variation among younger ages, but they get less oscillated in the ranges of middle-age and elderly. While the second and the third principal components for female show kinds of the opposite direction of each other.

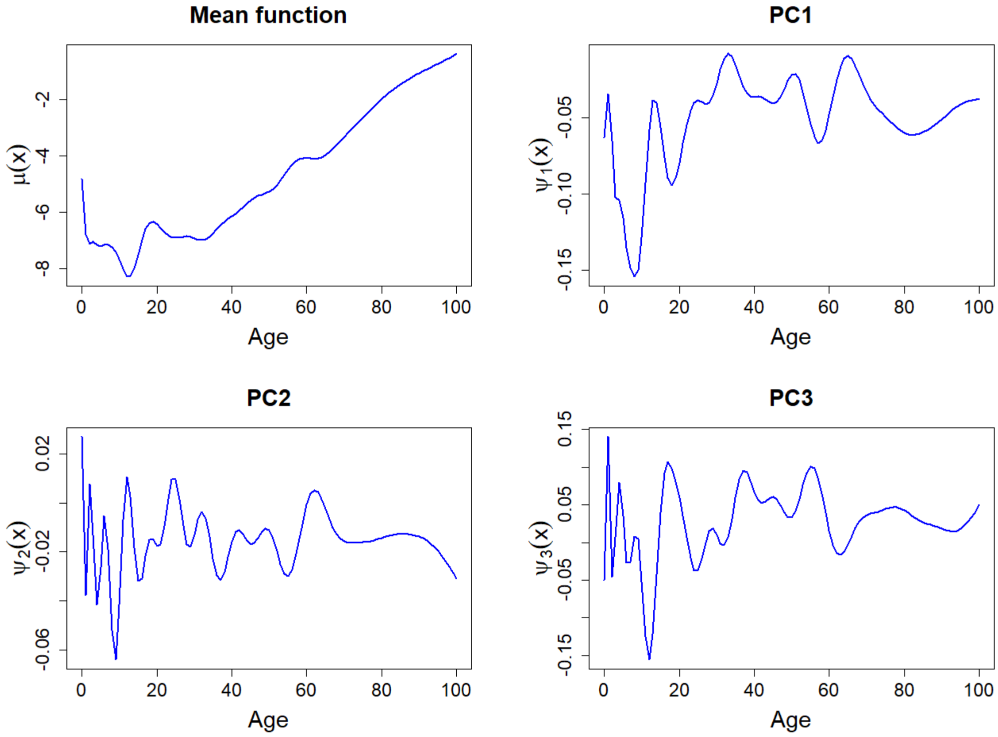

(a) Male

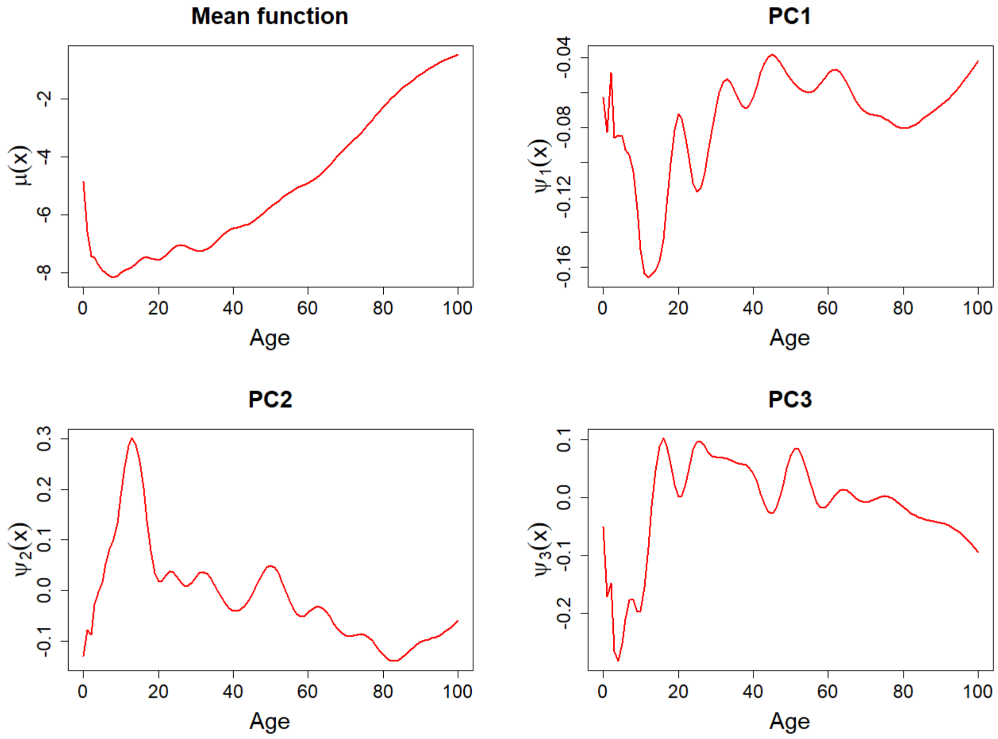

(b) Female

Figure 1. Estimated mean functions and the first three functional principal components for male and female mortality in Japan. (a) Male (b) Female.

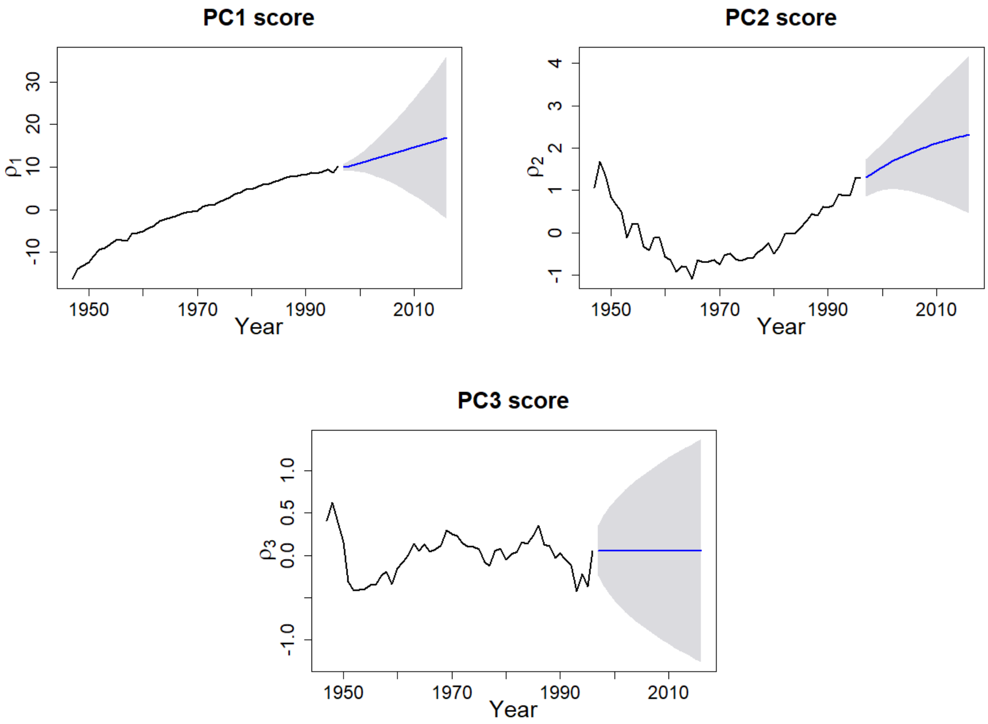

Figure 2. First three corresponding estimated scores of the PCs with the 20-years-ahead forecasts with the 95% prediction intervals (in grey).

2.2. *Sex-specific mortality modelling and forecasting by the coherent wMFPCA model*

2.2.1. *Estimated functional principal components and their corresponding scores for male and female using the coherent wMFPCA model*

In Figure 3, we display the estimated overall mean function $\hat{\mu}(x)$ and the first three estimated functional principal components $\{\hat{\phi}_k(x)\}_{k=1}^3$. The corresponding scores of the first three estimated functional principal components $\{\hat{\beta}_{t,k}\}_{k=1}^3$ are presented along with 20-years-ahead out-of-sample forecast means and 95% confidence intervals by ARIMA models in Figure 4. The first three common trend functional principal components capture 98%, 1.7% and 0.2% variations, respectively, and more than 99% of the variations in the age-specific total mortality overall among this dataset. Each functional principal component models different movements in mortality rates. $\hat{\phi}_1(x)$ primarily models the degree of variations in mortality among different age groups as we expect that the variance of mortality rates in young age groups is relatively larger than elderly groups. In contrast, $\hat{\phi}_2(x)$ and $\hat{\phi}_3(x)$ model the young adult age below 40 and differences between late teen and those over 60. From the first forecast common principal component scores, the declines of mortality rates seem to continue, and we do not spot any apparent pattern from the second and third estimated common principal component scores.

In Figure 5, we plot the estimated male and female deviation functions from the overall mean. It is obvious that the estimated male deviation function $\hat{\eta}^{\mathrm{M}}(x)$ is a positive function, while the female estimated deviation function $\hat{\eta}^{\mathrm{F}}(x)$ covers whole

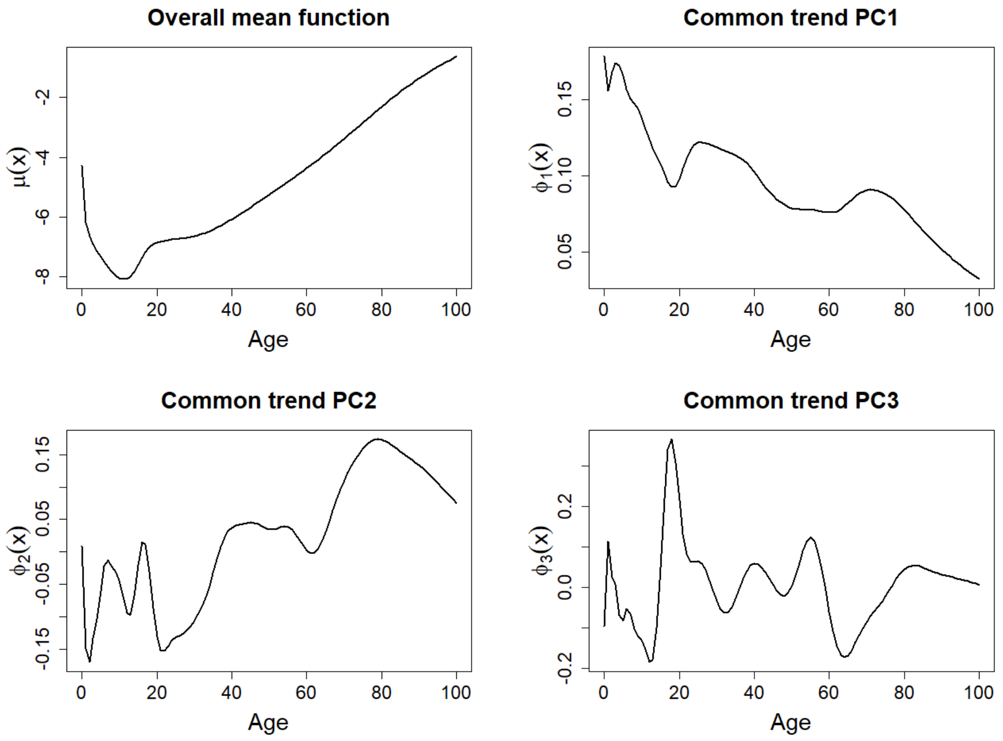

Figure 3. Estimated overall mean function and the first three estimated common trend functional principal components for the total mortality rates in Japan.

negative range over all ages. These indicate that the male mortality rates are in general higher than female's, and the difference reaches its peak at around age 20. All estimated male and female deviation functions from the overall mean behave like an upside-down reflection of each other and demonstrate completely reverse patterns. Figure 6 shows the first three estimated corresponding deviation trend functional principal component scores for male and female. From the forecasts of the three shared principal component scores among two genders, they seem to have a flat zero-convergent trend. It is, therefore, likely to achieve non-divergent forecasts between male and female subpopulations in the long term.

2.3. Forecast accuracy evaluation with comparisons to other existing methods

Figure 7 shows the historical mortality sex ratios (Male/Female) which are obtained from the observed male and female mortality rates from the year 1997 to the year 2016 alongside the 20-years-ahead forecasts of the mortality sex ratios from the year 1997 to the year 2016 by the non-coherent forecast methods - the independent FPCA model, the unweighted MFPCA model and the wMFPCA model and the coherent forecast methods - the Product-Ratio model, the weighted multilevel FPCA model and the coherent wMFPCA model using the observed mortality rates from the year 1947 to the year 1996. We can see that all the coherent forecasting methods exhibit a fairly one-to-one smooth pattern with the actual mortality sex ratio, and with much less fluctuation than the non-coherent forecast methods.

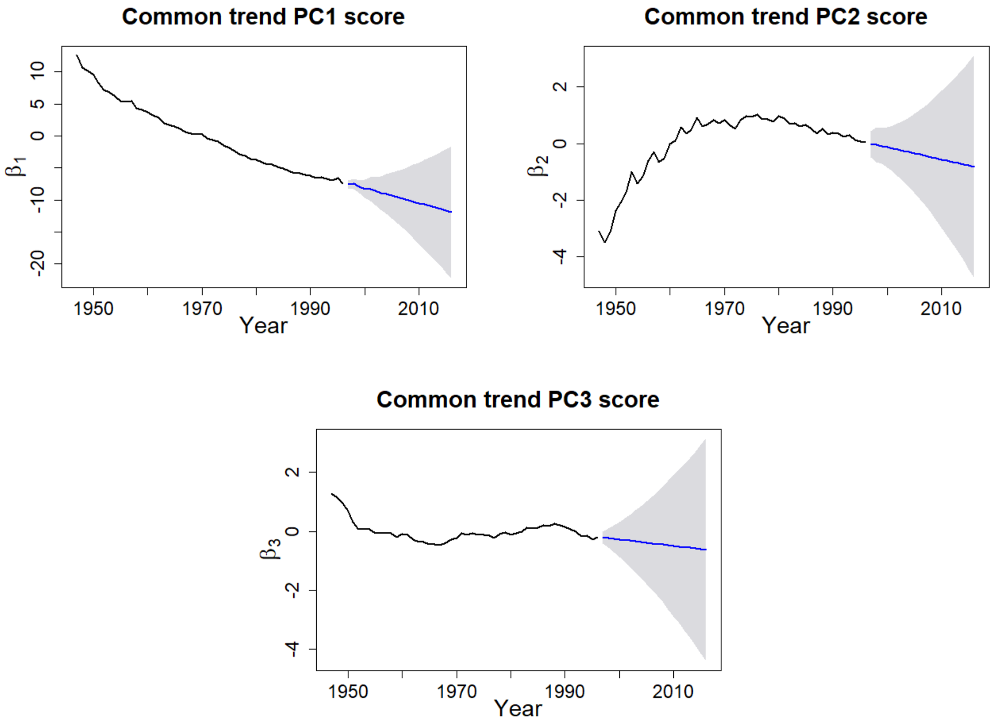

Figure 4. First three corresponding estimated PC scores of the common trend with 20-years-ahead forecasts and 95% prediction intervals (in grey).

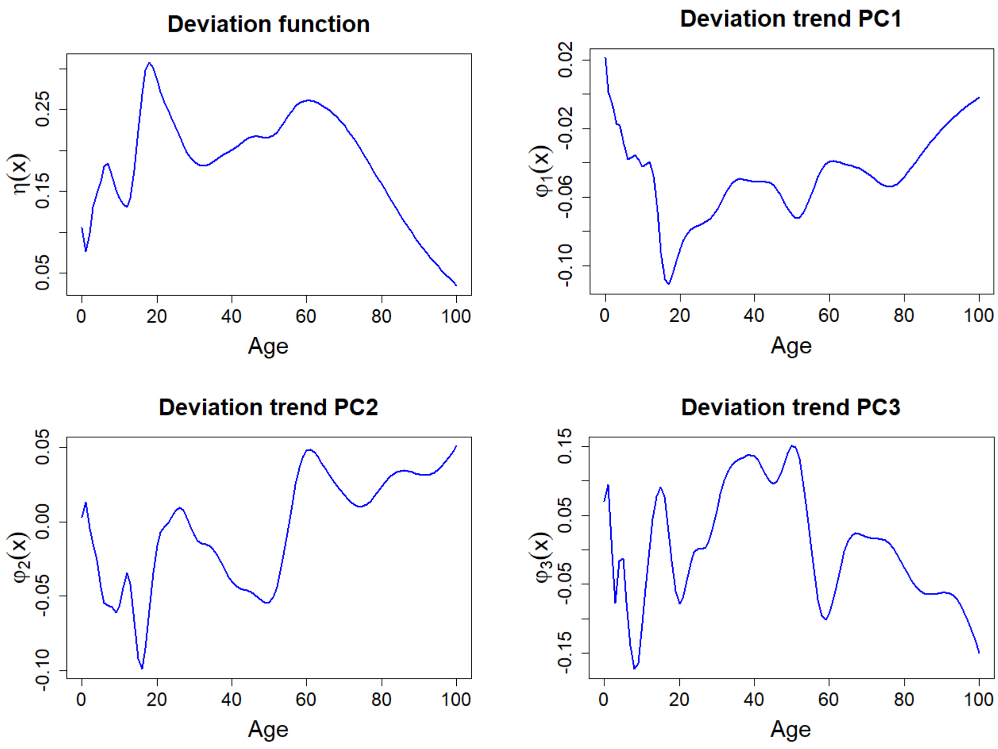

(a) Male deviation from the overall mean function

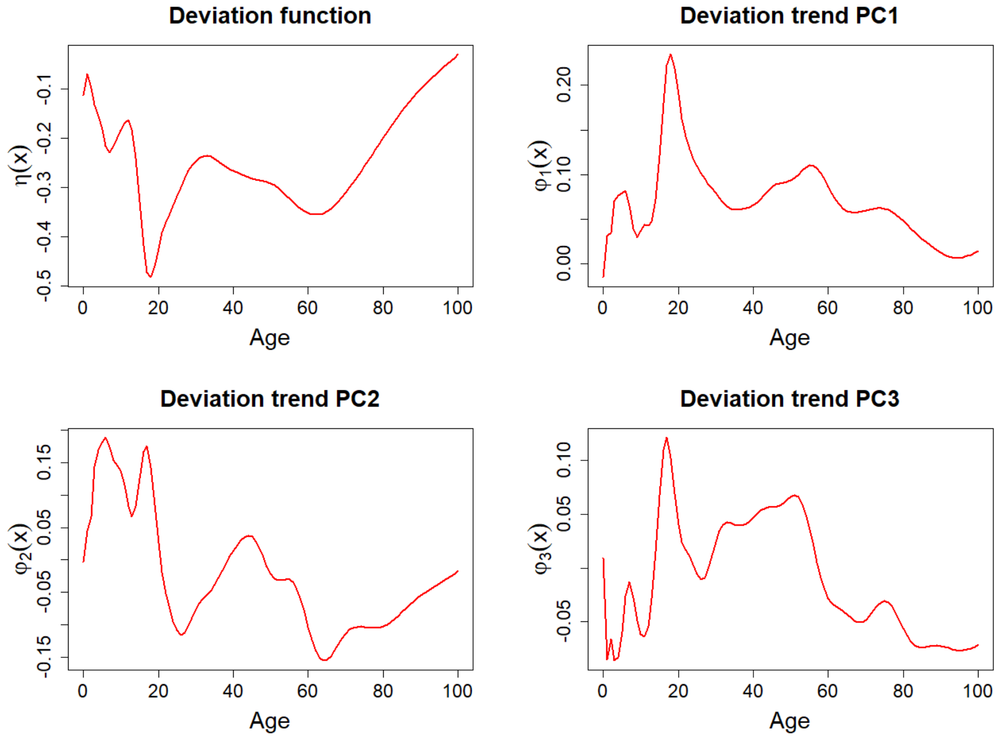

(b) Female deviation from the overall mean function

Figure 5. Estimated deviation functions from the overall mean function and the first three estimated deviation trend functional principal components for male and female mortality rates in Japan. (a) Male deviation from the overall mean function (b) Female deviation from the overall mean function.

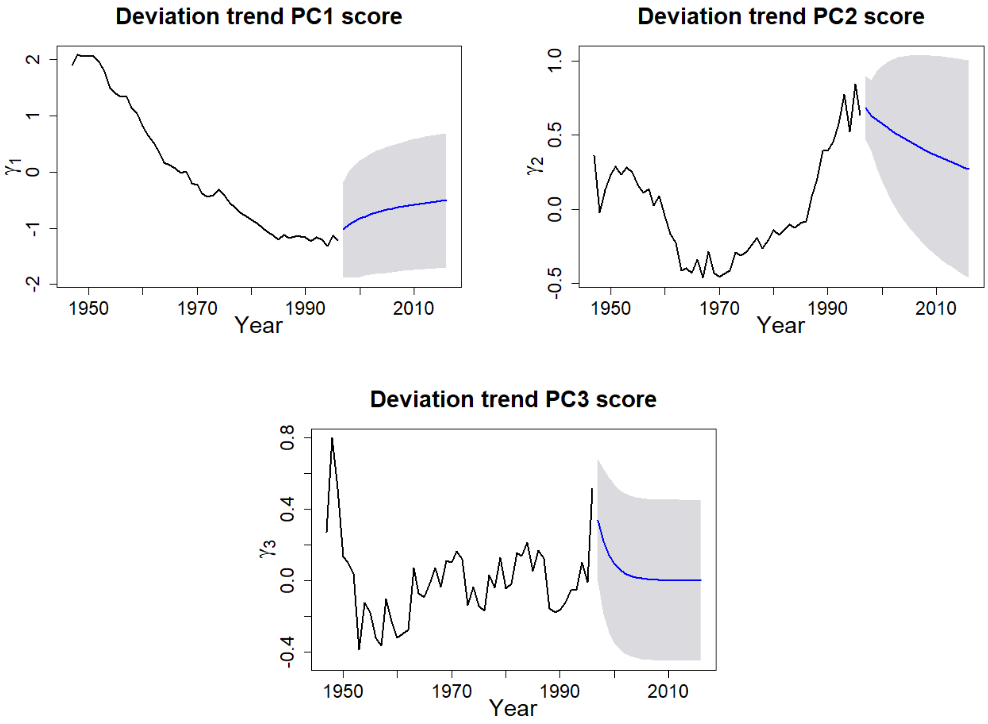

Figure 6. First three corresponding estimated PC scores of the deviation trend with the 20-years-ahead forecasts and the 95% prediction intervals (in grey).

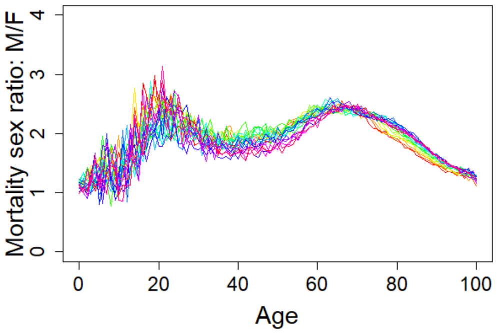

(a) Historical data

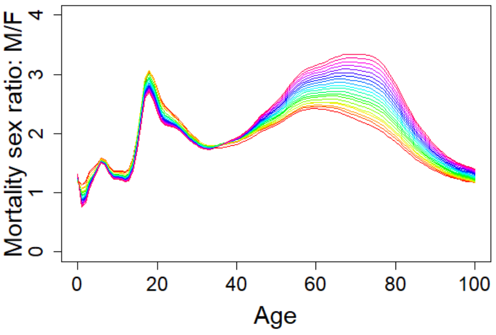

(b) Independent FPCA model

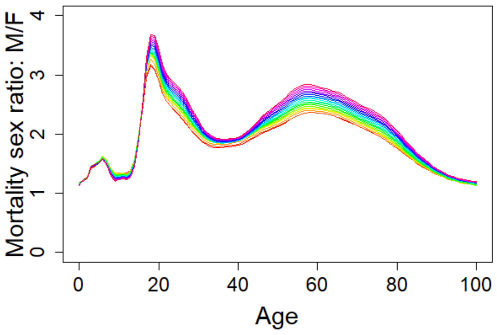

(c) Unweighted MFPCA model

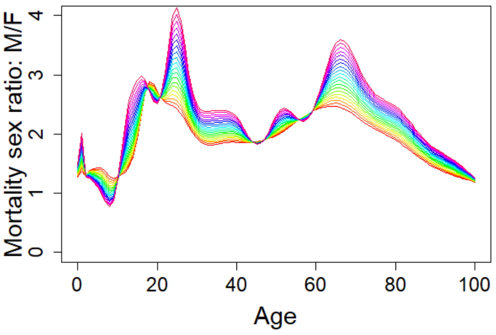

(d) wMFPCA model

Figure 7. Historical Japanese mortality sex ratio and 20-years-ahead forecasts of mortality sex ratios in Japan from the year 1997 to the year 2016 using the independent FPCA model, the unweighted MFPCA model, the wMFPCA model, the Product-Ratio model, the weighted multilevel FPCA model and the coherent wMFPCA model. (a) Historical data, (b) Independent FPCA model, (c) Unweighted MFPCA model, (d) wMFPCA model, (e) Product-Ratio model, (f) Weighted multilevel FPCA model, (g) Coherent wMFPCA model.

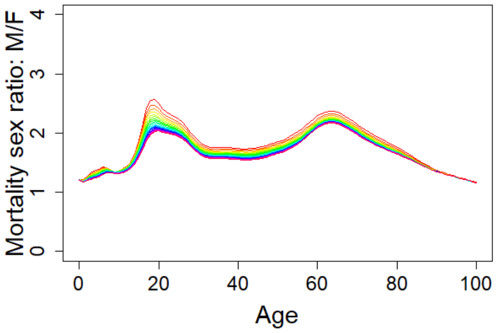

(e) Product-Ratio model

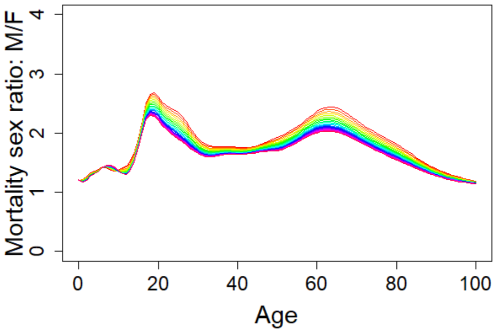

(f) Weighted multilevel FPCA model

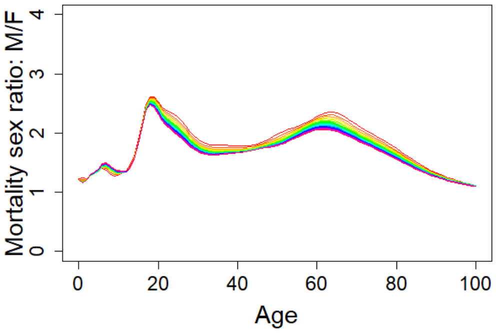

(g) Coherent wMFPCA model

Figure 7. Continued.

References

[1] S.M. Zemyan, *The Classical Theory of Integral Equations: A Concise Treatment*, Springer, New York 2012.
